# Quantifying Impacts of Biomass Pelletization on Fast Pyrolysis Using a Single-Particle Reactor, X‑ray Computed Tomography, and Computational Modeling

**DOI:** 10.1021/acs.energyfuels.5c04362

**Published:** 2025-11-20

**Authors:** Meagan F. Crowley, Reinhard Seiser, Mario Alejandro Sánchez Posada, Juan C. Maya, Farid Chejne, Hariswaran Sitaraman, Francois Usseglio-Viretta, Anne K. Starace, Peter N. Ciesielski

**Affiliations:** † 53405 National Renewable Energy Laboratory (NREL), Golden, Colorado 15013, United States; ‡ Grupo de Investigación ENERGEIA, Escuela de Ingeniería y Ciencias Básicas, Universidad EIA, Envigado 055428, Colombia; § Grupo de Investigación TAYEA, Departamento de Procesos y Energía, Facultad de Minas, 124148 Universidad Nacional de Colombia-Sede Medellín, Medellín 050034, Colombia

## Abstract

The pore structure and density of lignocellulosic feedstocks dictate intraparticle transport phenomena and thereby play an important role in thermochemical conversion processes such as fast pyrolysis for biofuel and biochemical production. Variations in microstructure are inherent from different biomass species and can be introduced by preprocessing techniques such as cutting and pelletization. Morphological changes also occur during conversion and lead to vastly different pore structures and behavior during pyrolysis, which impact required conversion times and product distributions. The current work presents a comprehensive comparison of fast pyrolysis of neat and pelletized pine feedstocks, which includes single-particle experiments, modeling, and 3D imaging by X-ray computed tomography (XCT). The particle-scale model included anisotropic heat and mass transport in a shrinking particle with pyrolysis reactions based on the CRECK mechanism with boundary conditions informed by reactor-scale simulations of the single-particle reactor. The models were validated by measurements of the temperature and mass loss from single-particle pyrolysis experiments of neat and pelletized pine. Quantitative analysis of XCT geometries revealed that pyrolytic conversion yielded chars with increased porosity and permeability compared to the unpyrolyzed materials, along with decreased tortuosity and anisotropy. Pelletization of the pine feedstock resulted in a much denser, less permeable material, which converted slower and produced more residual char after pyrolysis compared to neat pine. The results from particle modeling revealed that accounting for the dynamic and anisotropic heat and mass transport caused by differences in pore structure is critical to achieving agreement with experimental results. Overall, this study highlights the dramatic differences in conversion behavior imparted by pelletization and the importance of capturing microstructural attributes in computational models to guide the design and optimization of pyrolysis processes for specific biomass feedstocks.

## Graphical abstract

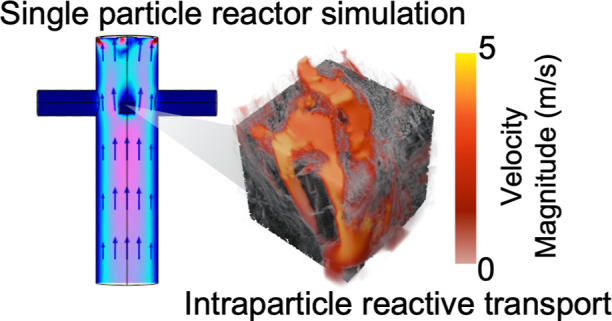

## 1 Introduction

Fast pyrolysis of lignocellulosic biomass offers a promising route to bolster production of renewable fuels and chemicals with favorable life cycle carbon emissions.^1^ A recent assessment sponsored by the U.S. Department of Energy, termed the “2023 Billion-Ton Report”, indicates that ample biomass feedstocks are available to supply the domestic bioeconomy; however, these will include a wide variety of sources, from agricultural and logging residues to municipal solid waste streams.^2^ Biomass feedstocks inherently exhibit a broad range of variable characteristics, such as density, biopolymer composition, and microstructure. Additionally, feedstock handling and preprocessing, such as comminution, drying, pelletization, and storage, can introduce further sources of variability.^3^ Ultimately, the performance of the pyrolysis process is dictated by a combination of feedstock characteristics, reactor configurations, and operating conditions. Developing a quantitative relationship between feedstock attributes and process variables is central to effective deployment, scaling, and optimization.

Experimentation and modeling of pyrolysis at the single-particle level have emerged as powerful tools for interrogating the impact of feedstock-specific effects.^4–6^ Careful single-particle experiments can decouple reactor-scale phenomena, such as bulk mixing and hydrodynamics, from processes that are dictated by material properties. However, a suitable computational framework must be employed to account for intraparticle transport phenomena.^5^

Thermogravimetric analysis (TGA) has been widely used to study the thermochemical conversion of biomass. Such studies are useful for determining intrinsic kinetic rates for very small particles, although intraparticle transport phenomena strongly influence the behavior of larger particles,^6^ which are more practical for commercial applications. Accurate parametrization of porous media transport models within biomass particles requires detailed characterization of the materials’ microstructure.

In this study, we coupled single-particle pyrolysis experiments with high-fidelity modeling and characterization to elucidate the impact of pelletization on softwood feedstocks subjected to fast pyrolysis conditions. A multiscale computational framework was developed to simulate pyrolysis of native and pelletized wood in NREL’s custom single-particle reactor (SPR), which is capable of producing rapid heating rates characteristic of fast pyrolysis. Reactor models were used to determine the local environment experienced by biomass in the reactor, which served as the boundary conditions for the particle-scale model. The microstructures of feedstock specimens and their resultant char were imaged by X-ray computed tomography (XCT). Quantitative analysis of the 3D reconstructions was used to calculate the intraparticle transport properties. These data, combined with the output from reactor-scale models, were used to parametrize detailed particle models, which considered complexities including dynamic, anisotropic transport properties and shrinkage, coupled to pyrolytic kinetic schemes. The predictions of these simulations were compared to measurements of mass loss from experiments performed under corresponding conditions.

## 2 Experimental

### 2.1 Feedstock Characterization

The particle samples were made of loblolly pine (*Pinus taeda*), an abundant feedstock in the Southeastern US, collected and prepared by Idaho National Laboratory (INL). Neat pine samples were obtained from a slice of a tree stem (170 mm diameter) and coring samples by using a plug cutter in the axial direction at various locations at a radius of 125 mm. The slice of the tree stem used for coring particle samples is shown in Figure 1. These samples (9.5 and 6 mm diameter and cut to lengths of 12 and 8 mm, respectively) are referred to as “cut pine”. The second type of feedstock was obtained by grinding up the stem of the tree into a powder and pelletizing it with a drum-type pellet mill. These samples are referred to as “pelletized pine”. Cut pine and pelletized pine chars were also collected after the pyrolysis experiments for microstructural characterization. The particles used for microstructural characterization are shown in Figure 2.

**Figure 1:**
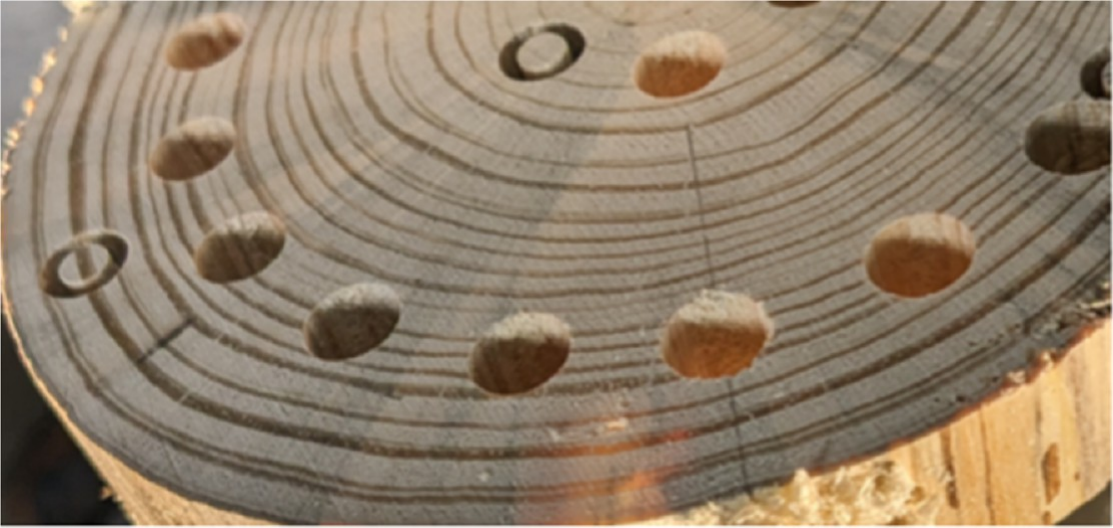
Slice of a tree stem used for coring of particle samples.

**Figure 2:**
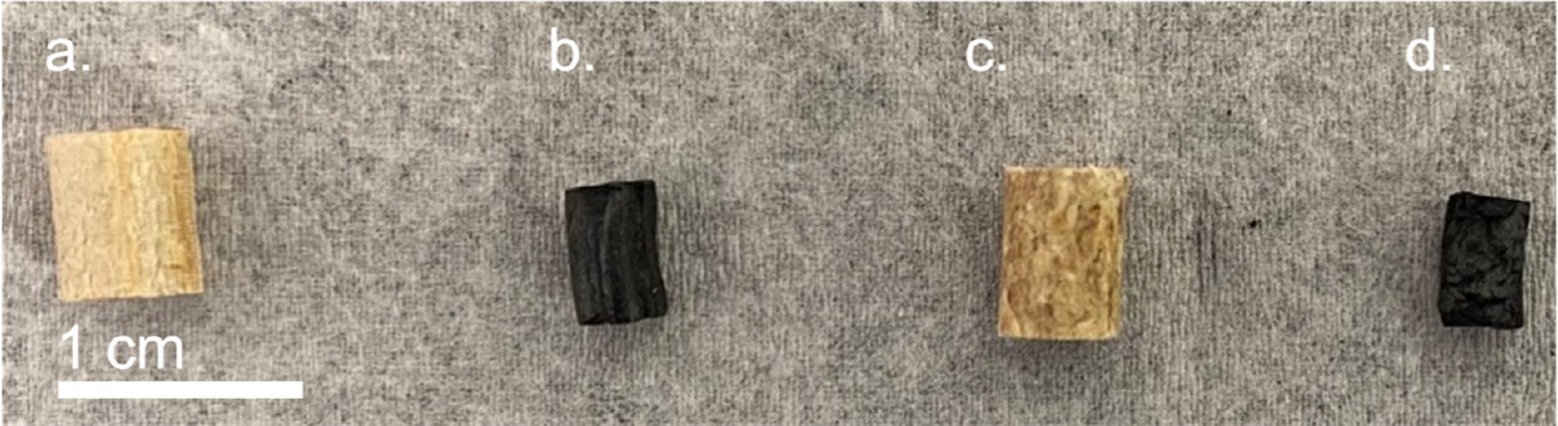
Optical images of representative loblolly pine samples used in this study: (a) cut pine, (b) cut pine char, (c) pelletized pine, and (d) pelletized pine char.

Ultimate and proximate analyses were completed on the feedstocks along with density measurements of each particle, as shown in the Supporting Information (Tables S1 and S2). The density of the pelletized pine was approximately twice that of the cut pine. The pelletized pine had slightly higher carbon, volatile matter, and ash content than the cut pine, while it had less moisture, nitrogen, and fixed carbon. Both feedstocks had roughly equivalent amounts of hydrogen.

### 2.2 Single-Particle Reactor Experiments

Pyrolysis experiments were conducted in a SPR that combines features of a macro TGA (thermogravimetric analysis, i.e., recording of weight loss) as well as gas collection, optical observation, and higher heating rates than typically available in commercial TGAs. Similar reactors have been designed and used by other research groups.^7–9^ A schematic of the SPR is shown in Figure 3. A reactor tube (35 mm ID) made of Inconel 625 is heated from the outside by a vertical three-zone tube furnace (Applied Test Systems, ATS—3210-2.00-10-24.25). Additionally, the main process flow (nitrogen) is preheated by heat-traced lines before it enters the reactor near the bottom. On top of the reactor, a platform holds a recording balance (Sartorius WZA224L) with an enclosure that is purged by helium. Helium, with its lower density, prevents heavier pyrolysis vapors migrating upward into the scale enclosure. Before the start of the experiment, the sample is loaded into a wire basket (made of 0.15 mm nichrome wire) hanging from the recording balance. A porous tube (Hastelloy C22) above the sample is used to evenly dilute the pyrolysis vapors to prevent condensation and reaction in heated downstream lines. The platform, including the scale, porous tube, and sample basket, can be lifted out of the reactor to enable loading of the sample and adjustment of the thermocouples near the sample. Before the start of the experiment, a water-cooled cup is moved to the top of the reactor to enclose the sample and prevent early reaction. The platform and cooling cup are lowered into the reactor with subsequent lowering of the water-cooled cup all the way to the bottom of the reactor, which marks the beginning of the experiment by exposing the sample instantaneously to a high temperature. This allows for a high heating rate (∼100 K/s for the first few seconds), as measured by the thermocouple external to the biomass sample. A high heating rate is important for the production of liquids (i.e., fast pyrolysis oil). While the somewhat larger particles (∼1 cm) exhibit smaller internal heating rates (as low as 5 K/s in the center) than the ground feedstocks employed in commercial fast pyrolysis reactors, understanding the heat conduction and mass transfer in larger particles is important to developing fundamental models that can be confidently applied to particles across a range of sizes.

**Figure 3:**
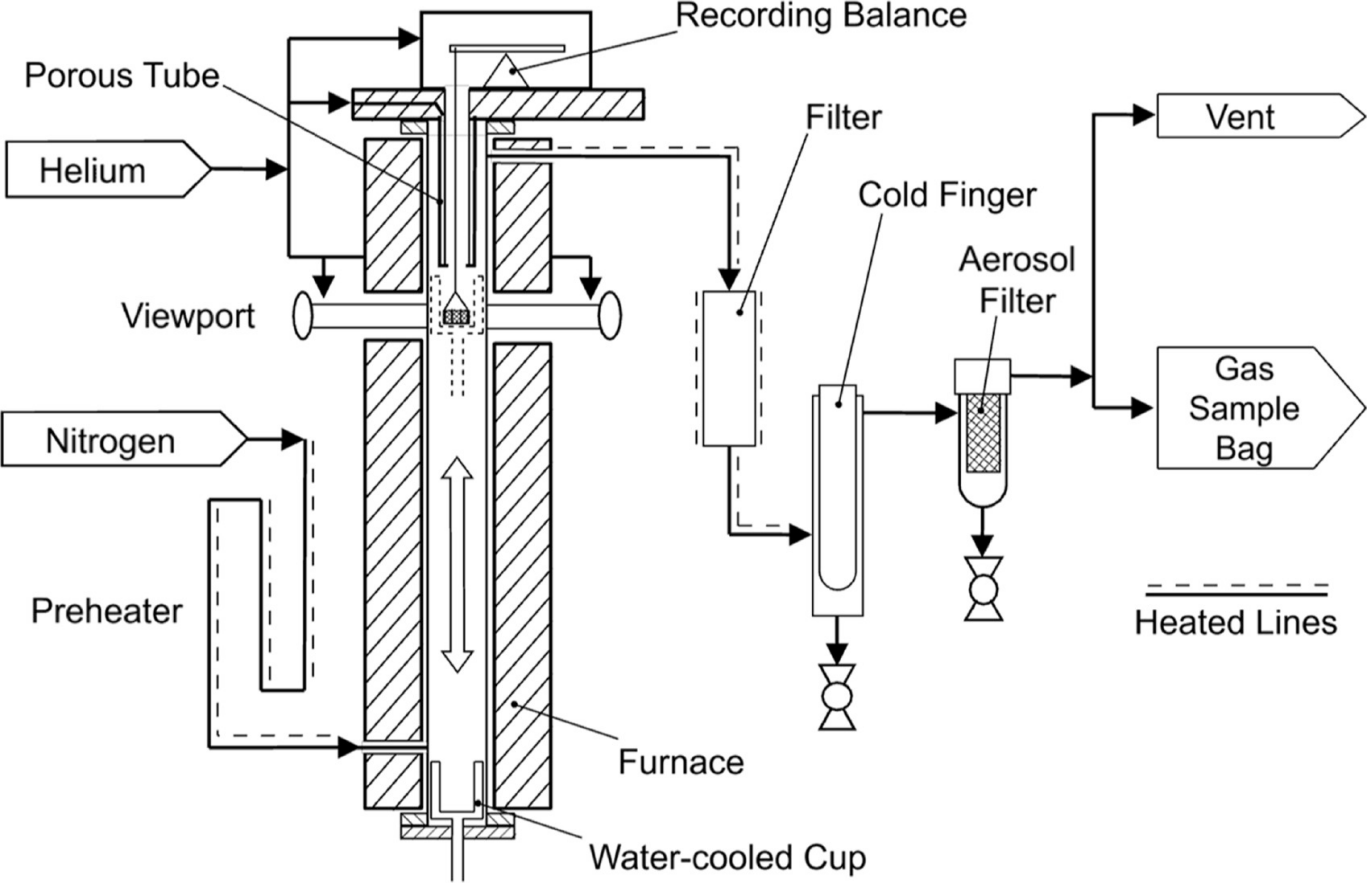
Schematic of SPR.

A relatively high nitrogen flow rate from the bottom (∼1 m/s) is another feature for providing a high convective heating rate for the biomass particle. Helium is used for purging the scale enclosure, porous dilutor tube, and side ports. The lower density of helium compared with nitrogen reduces the momentum of the streams from the side and top, and the slightly lower molar heat capacity provides gentle quenching. The residence time of the pyrolysis vapors before being quenched with helium is 25 ms, and the residence time of the diluted pyrolysis products in the downstream plumbing (heat traced at 400 °C) is 0.2 s.

Temperatures were measured with K-type thermocouples. The temperature of the furnace (outside the reactor tube) provided the nominal conditions (650 and 700 °C, respectively), and thermocouples inside the reactor measured the process conditions. The thermocouple below the basket (0.25 mm wire with a bead positioned 5 mm below the bottom of the sample and 8 mm from the center of the reactor) represents the free-stream temperature of the nitrogen flow. For two experiments on the 9.5 mm diameter cut pine at 650 °C, an additional thermocouple was placed inside the particle, with a 25 μm wire running inside a hole (0.21 mm diameter) drilled along the axis of the particle and the bead in the center of the particle. These thin thermocouple wires were connected to larger support wires (0.25 mm) outside the particle.

The mass loss of the particles over time during pyrolysis in the SPR was calculated from triplicate runs of cut pine and pelletized pine samples according to eq 1

1 $$mass\;loss=\frac{w_{t}}{w_{0}}$$

where *w*_*t*_ is the measured weight of the particle at time *t* and *w*_0_ is the initial weight of the particle. Weight measurements were recorded during pyrolysis in the SPR. Multiple weight-loss curves were averaged across weight values at each time step. In cases where there was a distinct shift of the curves in the time axis, averages were constructed diagonally across reaction curves constructed between characteristic curvature and inflection points. Error bands were always calculated at given time values by determining the standard deviations of the weights from the average weights and showing the ±2σ values relative to the average weight values.

Pyrolysis experiments on the pelletized pine samples measuring 6 mm diameter and 8 mm length were performed at 650 and 700 °C. For the cut pine samples, experiments were performed on particles of different sizes to investigate the impact on weight loss: 6 mm diameter, 8 mm length cylindrical particles and 9.5 mm diameter, 12 mm length cylindrical particles. The 9.5 mm diameter particles were pyrolyzed at two different temperatures, 650 and 700 °C.

### 2.3 X-ray Computed Tomography and Quantitative Structural Analysis

3D reconstructions of the microstructure of cut pine, pelletized pine, cut pine char, and pelletized pine char samples were collected using XCT on a Zeiss Xradia 520 Versa X-ray microscope at Colorado School of Mines. The X-ray source was set to emit at 40 kV voltage, 3 W power, and 75.5 μA current. The detector used the 4× objective and bin 1 setting for data collection. Samples were mounted to wooden dowels using double-sided tape to secure them for imaging. For each sample, 1601 images were collected at 0.22° increments with 15 s of exposure time at each angle as the sample rotated from 0 to 360° on the stage. 4 mm diameter by 8 mm length cylindrical regions at the center of cylindrical cut pine and pelletized pine particles measuring 8 mm in length and 6 mm in diameter were collected before pyrolysis with a resolution of 2.004 μm. Cut pine char and pelletized pine char samples were smaller after being pyrolyzed, measuring approximately 4 mm in diameter by 6 mm in length, and the full particles were captured in the 4 mm × 8 mm field of view for the scans.

Zeiss’ proprietary reconstruction software was used to export 3D reconstructions of each sample. The 3D reconstructions were then cropped to 1 mm^3^ sub-volumes, filtered, and segmented into solid and void phases using MATBOX,^10^ available at https://github.com/NREL/MATBOX_Microstructure_analysis_toolbox and Dragonfly software.^11^ A schematic of the workflow for XCT data collection is shown in Figure 4. The workflow was also tested on larger rectangular subvolumes measuring approximately 3 × 7 × 3 mm^3^, but the intensity variations within XCT scans and the large amount of memory required for analysis were prohibitive to proper segmentation.

**Figure 4:**
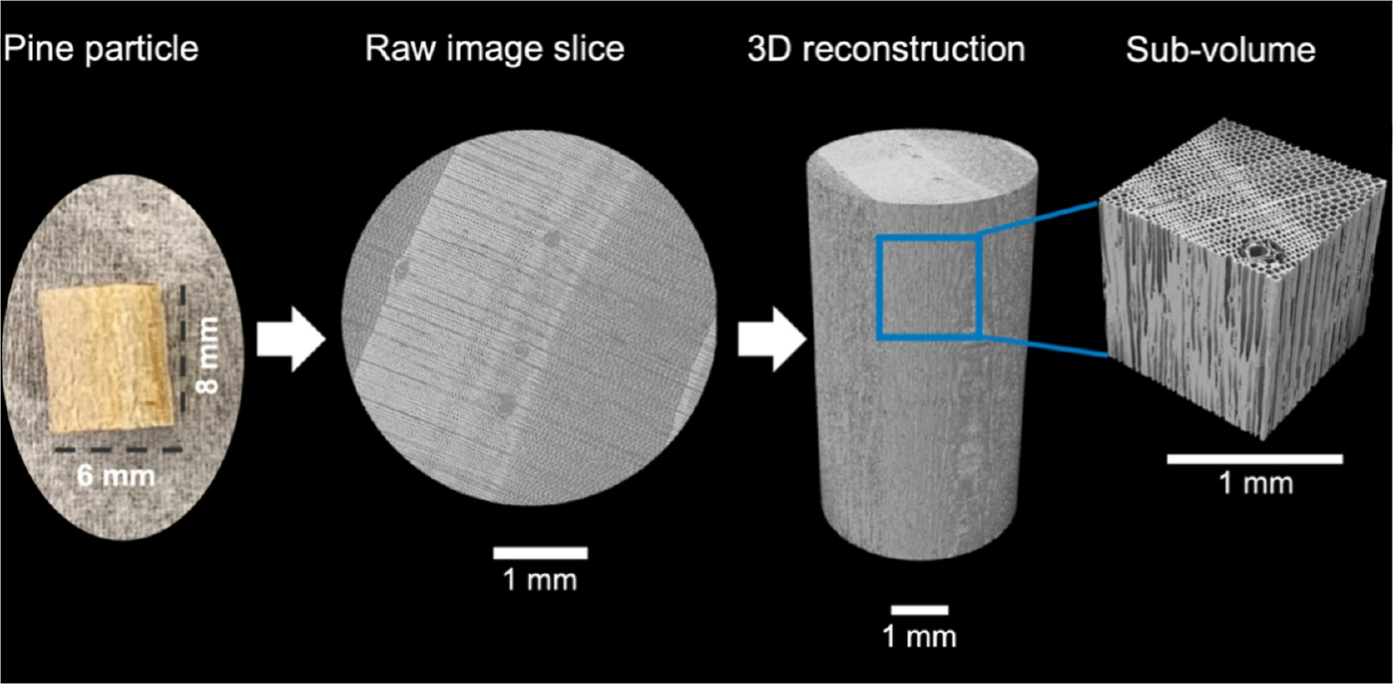
Schematic of XCT workflow. 6 mm by 8 mm cylindrical cut pine particles were imaged, and 3D geometries were reconstructed. One cubic millimeter subvolumes were extracted from the reconstructed geometries for microstructural analysis.

The sub-volumes were filtered and segmented into void and solid phases using Dragonfly software and MATBOX. The nonlocal means filter was used to reduce noise in the XCT data prior to segmentation via a global intensity threshold. The global threshold values for each sample were selected to produce porosities that were consistent with the measured densities reported in Table S2. That is, it was assumed the imaged field of view was representative of the volume fractions. For sub-volumes with a high amount of noise and low contrast, the process islands feature was used in Dragonfly to remove isolated solid regions smaller than 100 pixels in each of the 2D slices. The porosity and tortuosity were calculated using MATBOX microstructural analysis and TauFactor [https://github.com/tldr-group/taufactor]. Detailed descriptions of the algorithms used to calculate geometric properties can be found in previous publications.^10,12,13^ Briefly, the porosity, ε_G_, was calculated by averaging the number of voxels assigned to the void phase by the total number of voxels, *N*, in the segmented sub-volume, according to eq 2

2 $$\varepsilon_{G}=\frac{1}{N}\sum_{i=0}^{N}v\left(i\right),,with\;v\left(i\right)=\{\begin{array}{l} 1\;\text{if}\;v\left(i\right)\in of\;the\;void\;phase\\ 0\;\text{if}\;v\left(i\right)\notin of\;the\;void\;phase \end{array}$$

The tortuosity factors, τ_*i*_, were calculated for the pore domain using a finite-difference-based approach with fixed Dirichlet boundary conditions. The Laplace equation was solved in each direction within the sub-volume and the effective diffusion coefficient, *D*_eff,*i*_, was calculated from a 1D Fick’s law approximation considering a unit value bulk diffusivity in the pore, i.e., *D*_bulk_ = 1. The tortuosity τ_*i*_ and the Bruggeman exponent *p*_*i*_ in the *i*th direction were then solved from the ratio between the bulk and effective diffusion coefficients along with the porosity according to eqs 3a and 3b. Unlike the tortuosity factor, the Bruggeman exponent is not a function of the porosity and indicates the contributions of the particle shape and spatial distribution on the effective diffusivity

3a $$\tau_{i}=\varepsilon_{G}\frac{D_{bulk}}{D_{eff,i}}$$

3b $$\frac{D_{eff,i}}{D_{bulk}}={\varepsilon_{G}}^{p_{i}}\;then\;\tau_{i}={\varepsilon_{G}}^{1-p_{i}}$$

### 2.4 Computational Modeling

#### 2.4.1 Determination of Permeability via Intraparticle Flow Simulations

The permeability tensors of cut pine, pelletized pine, pine char, and pelletized pine char were calculated from computational fluid dynamics (CFD) simulations of air flow through the XCT reconstructions of each sample using our open-source fluid dynamics solver, Mesoflow,^14^ available at https://github.com/NREL/mesoflow. Mesoflow leverages the Cartesian adaptive mesh management library, AmReX,^15^ which has been used in several recent studies with applications to multiphase flows,^12,16–18^ combustion,^19^ and astrophysical flows.^20^ Additionally, Mesoflow utilizes an immersed boundary method to resolve complex pore structures from XCT data sets with high resolution and simulate intraparticle momentum transport.

To perform the intraparticle flow simulations, the XCT data sets were imported directly into Mesoflow as voxelated files of intensities and converted into solid and void volume fractions on a Cartesian grid. A threshold value was implemented for each XCT data set to separate the geometry into solid and void phases. Hyperbolic and viscous fluxes were calculated at fluid–solid interfaces by imposing no-slip conditions interpolated onto the Cartesian grid. A modified advection upwind splitting method (AUSM) scheme^21^ for low Mach number flows and a second-order reconstruction and limiting scheme^22^ were used for hyperbolic flux discretization. To discretize the viscous flux, a second-order central differencing scheme was used. Finally, a second-order explicit Runge–Kutta scheme was used for time advancing in the simulations.

To calculate the principal components of the permeability tensor, intraparticle fluid flow simulations were performed using the XCT geometries for each sample as the simulation domain. Three separate simulations were performed for each sample to extract principal permeabilities in the *x*, *y*, and *z* directions. For each simulation, the boundaries along the direction of flow were initialized with a pressure inlet and outlet and a 1 × 10^4^ Pa pressure drop across the XCT geometry in the specified direction. All other boundaries were treated as walls with no-flux boundary conditions. Simulations were run to steady state, as determined by diminishing density and momentum residuals and constant exit velocity. The velocity field was solved via the continuity equation and time-dependent compressible Navier–Stokes equation in the direction of interest according to eqs 4 and 5, respectively:

4 $$\frac{\partial \rho }{\partial t}+\frac{\partial \left(\rho U_{i}\right)}{\partial x_{i}}=0$$

5 $$\frac{\partial \left(\rho U_{i}\right)}{\partial t}+\frac{\partial }{\partial x_{j}}\left(\rho U_{i}U_{j}\right)=-\frac{\partial P}{\partial x_{i}}+\frac{\partial \tau_{ij}}{\partial x_{j}}$$

where ρ is the fluid density calculated using the ideal gas law, *U*_*i*_ is the component of the velocity vector in the *i*th direction, *x*_*i*_ is the spatial coordinate in the *i*th direction, *P* is the fluid pressure, and τ_*ij*_ is the respective component of the viscous stress tensor. The ideal gas and Newtonian fluid assumptions, according to eqs 6 and 7, respectively, were utilized to solve for the velocity field.

6 $$P=\rho R_{s}T$$

7 $$\tau_{ij}=\mu \left(\frac{\partial U_{j}}{\partial x_{i}}+\frac{\partial U_{i}}{\partial x_{j}}\right)-\frac{2}{3}\mu \frac{\partial U_{k}}{\partial x_{k}}\delta_{ij}$$

where μ is the viscosity, *R*_s_ is the specific gas constant for air used in these simulations, *T* is the temperature, set to 293.15 K in these simulations, and δ_*ij*_ is the Kronecker delta function.

The permeability in the *i*th direction, κ_*i*_, was then calculated from Darcy’s law using the fluid viscosity μ, surface-averaged normal fluid velocity $\left(\overset{®}{U_{i}}\right)$ and the pressure drop along the *i*th direction (Δ*P*_*i*_) divided by the length of the domain in the *i*th direction (*L*_*i*_), according to eq 8:

8 $$\kappa_{i}=\mu \overset{®}{U_{i}\;}{\left(\frac{\Delta P_{i}}{L_{i}}\right)}^{-1}$$

Mesoflow simulations were performed on the NREL Kestrel supercomputer, using 4096 processors in parallel for 250,000 to 1,200,000 total compute hours per simulation.

#### 2.4.2 Reactor Modeling

A CFD model of the SPR was developed in COMSOL Multiphysics v.6.1 ^23^ to investigate the temperature near the sample over time and extract the boundary conditions for use in particle models. The 3D reactor geometry was modeled with a horizontally oriented cylindrical biomass sample located at the position of the sample basket. A point probe was defined at the position of the thermocouple used in experiments, 5 mm below the sample and 8 mm from the center of the reactor. The geometry of the reactor, the position of the thermocouple used to identify boundary conditions for the particle-scale model, and the location and orientation of the particle are shown in Figure 5.

**Figure 5:**
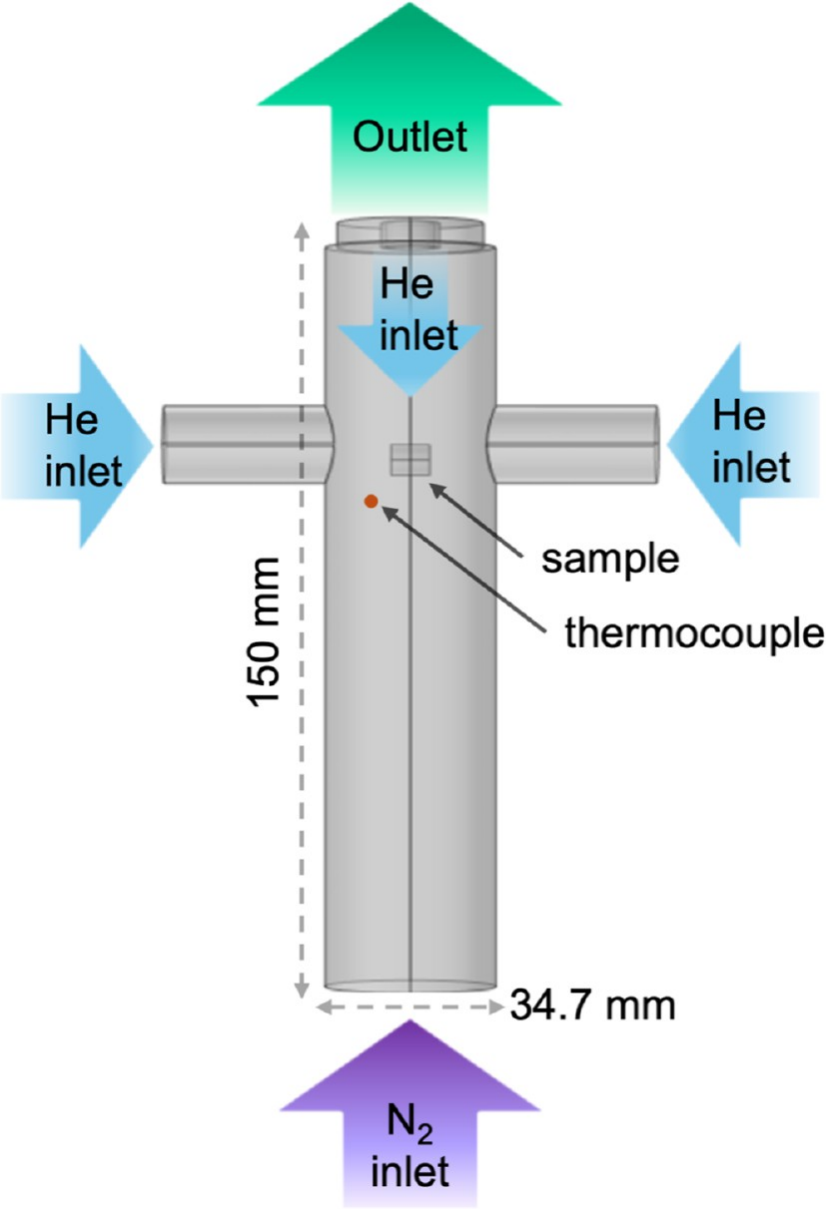
SPR reactor model geometry, position of the thermocouple used to identify temperature boundary conditions in particle models, and location and orientation of the sample developed in COMSOL.

The laminar flow, heat transfer in solids and fluids with porous medium, and surface-to-surface radiation modules were used to model the time-dependent mass, heat, and momentum transport in the reactor. Additionally, multi-physics couplings for non-isothermal flow and heat transfer with surface-to-surface radiation were included. Properties of the cut pine and pelletized pine samples determined from microstructural analysis and Mesoflow simulations, including porosity, density, and permeability, were included in the model to capture intraparticle heat and momentum transport. For model stability and efficiency, pyrolysis reactions were not incorporated into the reactor model. A wall temperature boundary condition was enforced at the furnace temperature, which was measured over time using the SPR experiment. An inlet boundary condition was implemented at the bottom of the reactor, where nitrogen gas entered with a volumetric flow rate of 0.001 m^3^/s. Additionally, helium gas inlets were implemented at the extremities of the two side tubes of the reactor with volumetric flow rates of 2.73 × 10^–8^ m^3^/s. Helium flow was also introduced at the top of the reactor with a volumetric flow rate of 3.64 × 10^–5^ m^3^/s. The outlet was enforced in an annular ring at the top of the reactor. The walls of the reactor were treated with no-slip boundary conditions. Properties of the model, including operating conditions, geometric descriptors, and transport properties, are included in Table 1.

**Table 1:** Thermophysical Properties Used in Reactor-Scale Models^a^

property (symbol)	value	description
*r*_tube_	0.01735	radius of main reactor tube (m)
*L*_tube_	0.150	length of main reactor tube (m)
*P*_std_	101325	standard pressure (Pa) for SLPM
*T*_std_	273.15	standard temperature (K) for SLPM
*P*_0_	101325	initial reactor pressure (Pa)
*T*_inlet_	843.29	bottom gas inlet temperature (K)
*T*_He_	298.15	side and top helium gas inlet temperature (K)
*T*_amb_	298.15	ambient temperature (K)
*T*_wall_	923.15	approximate reactor wall temperature (K), the actual temperature used in simulations was taken from experimental measurements over time
$U_{in,N_{2}}$	20	volumetric flow rate of nitrogen through the bottom inlet, SLPM
*U*_inside,He_	0.15	volumetric flow rate of helium through side inlets, SLPM
*U*_intop,He_	2	volumetric flow rate of helium through top inlet, SLPM
μ_N_2__	1.772 × 10^–6^ + 6.274 × 10^–8^ × *T* – 3.473 × 10^–8^ × *T* ^2^ + 1.012 × 10^–14^ *T* ^3^	temperature-dependent viscosity of nitrogen gas (from COMSOL v6.1 material library)
*r*_cutpine_	0.00315	radius of cut pine particle (m)
*r*_pelletizedpine_	0.00294	radius of pelletized pine particle (m)
*L*_pelletizedpine_	0.00817	length of pelletized pine particle (m)
*L*_cutpine_	0.00786	length of cut pine particle (m)
ρ_cutpine_	618.58	density of cut pine particle, kg/m^3^, measured from mass and volume of sample, assuming a perfect cylinder
ρ_pelletizedpine_	1239.24	density of pelletized pine particle, kg/m^3^, measured from mass and volume of sample, assuming a perfect cylinder
*C*_p_	2300	heat capacity of the biomass particle, J/(kg·K) from ref 25
*C*_p,f_	1088.221 – 0.366 × *T* + 7.887 × 10^–4^ × *T* ^2^ – 3.749 × 10^–7^ × *T* ^3^ + 3.176 × 10^–11^ × *T* ^4^	temperature-dependent heat capacity of nitrogen gas, J/(kg·K) from COMSOL v6.1 material library
*k*_s_	0.3	thermal conductivity of the solid biomass particle, W/(m·K) from ref 25
*k*_f_	2.58 × 10^–2^	thermal conductivity of the fluid, W/(m·K) from ref 25
ε_SE,wall_	0.35	surface emissivity of the reactor walls
ε_SE,part_	0.91	surface emissivity of the biomass particle
*n*	1	refractive index

a See Table S3 in the Supporting Information for additional nomenclature definitions.

The conservation equations solved in the reactor-scale simulations, similar to the intraparticle flow model, include the time-dependent Navier–Stokes and continuity equations, assuming laminar flow of a compressible Newtonian fluid with low Reynolds number (<2000) and low Mach number (<0.3) according to eqs 9–11:

9 $$\rho \frac{\partial \vec{U}}{\partial t}+\rho \left(\nabla \cdot \vec{U}\right)\vec{U}=\nabla \cdot \left[-PI+K\right]+\rho \vec{g}$$

10 $$\frac{\partial \rho }{\partial t}+\nabla \cdot \left(\rho \vec{U}\right)=0$$

11 $$K=\mu \left(\nabla \mathrm{U⃗}+{\left(\nabla \mathrm{U⃗}\right)}^{T}\right)-\frac{2}{3}\mu \left(\nabla \cdot \vec{U}\right)I$$

where ρis the density of the fluid, $\vec{U}$ is the fluid velocity vector, *P* is the pressure of the fluid, ***I*** is the identity tensor, ***K*** is the viscous stress tensor given by eq 11, and

$$\overset{⇀}{g}$$

***g*** is the acceleration due to gravity.

The particle was treated as a porous medium in the model, and the above equations were adjusted to consider momentum transport through porous media in the particle domain according to eqs 12–14

12 $$\frac{1}{\varepsilon }\rho \frac{\partial \vec{U}}{\partial t}+\frac{1}{\varepsilon }\rho \left(\vec{U}\cdot \nabla \right)\vec{U}\frac{1}{\varepsilon }=\nabla \cdot \left[-PI+K\right]-\left(\mu \kappa^{-1}+\beta \rho \left|\vec{U}\right|+\frac{Q_{m}}{\varepsilon^{2}}\right)\vec{U}$$

13 $$\frac{\partial \varepsilon \rho }{\partial t}+\nabla \cdot \left(\rho \vec{U}\right)=0$$

14 $$K=\mu \frac{1}{\varepsilon }\left(\nabla \vec{U}+{\left(\nabla \vec{U}\right)}^{T}\right)-\frac{2}{3}\mu \frac{1}{\varepsilon }\left(\nabla \cdot \vec{U}\right)I$$

where ε is the porosity of the particle calculated from microstructural analysis of XCT geometries, ρ is the density of the fluid phase, $\vec{U}$ is the velocity vector, *t* is time, ***I*** is the identity matrix, ***K*** is the viscous stress tensor assuming a compressible Newtonian fluid, μ is the viscosity of the fluid, κ is the permeability of the particle calculated from Mesoflow simulations, β is the isothermal compressibility coefficient, and *Q*_m_ is the volumetric mass source term.

Additionally, for heat transfer in the fluid domain, the general heat equation at constant pressure is solved, assuming Fourier’s law for the heat flux and the ideal gas law for the fluid density according to eqs 15–17

15 $$\rho C_{p}\frac{\partial T}{\partial t}+\rho C_{p}\vec{U}\cdot \nabla T+\nabla \cdot \vec{q}=Q+Q_{vd}$$

16 $$\vec{q}=-k\nabla T$$

17 $$\rho =\frac{P_{A}}{R_{s}T}$$

where *C*_p_ is the heat capacity at constant pressure, *T* is the temperature, $\vec{q}$ is the heat flux vector, *Q* is the heat source term, *Q*_vd_ is the energy transferred via viscous dissipation, *k* is the thermal conductivity, *R*_s_ is the specific gas constant, and *P*_A_ is the absolute pressure.

In the particle domain, the above equations are adjusted to solve for heat transfer within porous media according to eqs 18–21

18 $${\left({\rho C}_{p}\right)}_{eff}\frac{\partial T}{\partial t}+\rho_{f}C_{p,f}\vec{U}\cdot \nabla T+\nabla \cdot \vec{q}=Q+Q_{vd}$$

19 $$\vec{q}=-k_{eff}\nabla T$$

$${\left({\rho C}_{p}\right)}_{eff}=\varepsilon \rho_{f}C_{p,f}+\theta_{s}\rho_{s}C_{p,s}+\theta_{imf}\rho_{imf}C_{p,imf}$$ 20

$$k_{eff}=\varepsilon k_{f}+\theta_{s}k_{s}+\theta_{imf}k_{imf}+k_{disp}$$ 21

where *C*_p,eff_ is the effective heat capacity of the porous media, *C*_p,f_ is the heat capacity of the fluid, ρ_f_ is the density of the fluid, *k*_eff_ is the effective thermal conductivity of the porous media, *k*_f_, is the thermal conductivity of the fluid phase, ε is the porosity of the particle calculated from microstructural analysis of XCT geometries, θ_s_ is the solid volume fraction in the particle, *C*_p,s_ is the heat capacity of the solid, ρ_s_ is the density of the solid, θ_imf_, ρ_imf_, *k*_imf_, and *C*_p,imf_ are the volume fraction, density, thermal conductivity, and heat capacity of the immobile fluid within the particle, and *k*_disp_ is the dispersive thermal conductivity due to hydrodynamic mixing of fluid in the porous particle.

Additionally, radiative heat transfer, which is relevant at high pyrolysis temperatures,^24^ was implemented to model surface radiation between the reactor walls and the biomass particle. The radiative flux *J*, is given by eq 22, where ε_SE_ is the surface emissivity, *e*_b_(*T*) is the blackbody hemispherical total emissive power calculated using the Stefan–Boltzmann law from eq 23, and the total incoming radiative heat flux *G* is given by the sum of external, *G*_ext_, ambient, *G*_amb_, and mutual irradiation *G*_m_, as shown in eq 24. The calculation of the ambient radiative heat flux is given by eq 25, where *F*_amb_ is defined as the fraction of the surroundings that is not covered by other boundaries, σ is the Stefan–Boltzmann constant, *T*_amb_ is the ambient temperature, *T* is the temperature of the radiating material, and *n* is the refractive index.

22 $$J=\varepsilon_{SE}e_{b}\left(T\right)+\left(1-\varepsilon_{SE}\right)G$$

23 $$e_{b}\left(T\right)=n^{2}\sigma T^{4}$$

24 $$G=G_{m}+G_{amb}+G_{ext}$$

25 $$G_{amb}=F_{amb}e_{b}\left(T_{amb}\right)$$

The thermophysical properties used in reactor-scale models are reported in Table 1.

#### 2.4.3 Single-Particle Modeling

Multiscale modeling of biomass pellet pyrolysis provides crucial guidance to establish optimal operating conditions and select appropriate reactor sizes. To ensure the accuracy of these predictions, particle-scale models must incorporate critical phenomena and properties such as particle geometry and anisotropy, intraparticle mass and heat transfer, and detailed reaction mechanisms, including biomass pseudo-components.^26^

The developed model investigates the effect of the transport properties on particle conversion and product yields during the pyrolysis of biomass pellets. The model describes the pyrolysis of a shrinking cylindrical particle exhibiting anisotropic properties, incorporating intraparticle heat and mass transfer, along with the CRECK pyrolysis reaction mechanism with biomass pseudo-components and the generation of liquid intermediates.^27^ Model predictions were validated through comparison to experimental data obtained from the pyrolysis of individual cut pine and pelletized pine samples.

Previous studies on biomass fast pyrolysis modeling have emphasized the critical role of intraparticle heat and mass transfer. Through nondimensional analysis, Anca-Couce and Zobel^28^ found values of 3.2 and 10 of the Biot number and Pyrolysis number for 10 mm particles within fluidized-bed reactors at 800 K, highlighting the importance of temperature gradients and mass-transport phenomena within the particle. Likewise, Bharadwaj et al.^29^ demonstrated that incorporating both mass transfer and intraparticle heat and mass transport is essential for accurately predicting the pyrolysis rate of millimeter-sized particles.

Experimental and theoretical studies have demonstrated that particle morphology and anisotropy significantly affect drying, heating, and mass-loss kinetics during biomass pyrolysis.^6,30–34^ Lu et al.^32^ predicted that particle shape influences both overall conversion and the distribution of product yields: near-spherical particles generate fewer volatiles and more tars compared to aspherical particles of equivalent mass under identical conditions. Similarly, Ciesielski et al.^6^ showed that a spherical geometry heats slower than other shapes of the same volume due to its low surface-area-to-volume ratio, indicating that accurate geometric representation is crucial for reliable simulation outcomes.

Numerous particle-scale models for fast biomass pyrolysis incorporating mass and heat transport have been proposed;^28,31–47^ while the most recent consider mass and energy diffusive and convective transport, particle shrinking, and moisture evaporation, only a few of them consider simultaneously the effects of particle shape and anisotropy.^31,33,34,36,41,45^

The developed model enables a detailed analysis of the influence of particle characteristics and transport phenomena on the fast pyrolysis behavior of cylindrical anisotropic pellets. The model incorporates a comprehensive CRECK kinetic mechanism, which accounts for biomass pseudo-components and the formation of intermediate liquid species.^27^

##### 2.4.3.1 Model Considerations

Based on the previously discussed phenomena, the following assumptions are made:

- the model considers a porous, cylindrical biomass particle (Figure 6) undergoing anisotropic shrinkage in both radial and axial directions. Local porosity is dynamically calculated based on the spatial concentration of gas, liquid, and solid phases:

26 $$\varepsilon_{s}=\frac{C_{bm}}{\rho_{bm}}+\frac{C_{c}}{\rho_{c}}$$

27 $$\varepsilon_{L}=\frac{C_{MT}}{\rho_{MT}}+\frac{C_{H_{2}O,L}}{\rho_{H_{2}O,L}}$$

28 $$\varepsilon_{G}=1-\varepsilon_{S}-\varepsilon_{L}$$

where ε designates the volume fraction of solid (s), liquid (L), or gas phases (G), ε_G_ designates the local porosity, *C* designates local concentration, and ρ designates the density of the biomass (bm), char (c), metaplastic (MT), and liquid water (H_2_O,L) species.

- The model accounts for both diffusive and convective mass and heat transport along radial and axial directions. Direction-dependent transport properties of biomass and char, such as thermal conductivity and permeability, are incorporated. Heat transfer due to species diffusion is assumed to be negligible. Local thermal equilibrium is assumed among the solid, gas, and metaplastic phases. The metaplastic phase is treated as stationary, given its low mobility relative to permanent gases and volatiles.
- The reaction scheme implemented corresponds to the CRECK mechanism, which incorporates biomass pseudo-components and accounts for the formation of liquid intermediates (metaplastics).^27^ The model includes moisture evaporation and the intraparticle transport of water vapor. Additionally, it considers secondary homogeneous and heterogeneous reactions such as volatile cracking and charring occurring within the particle following.^34^
- Thermal properties are modeled as functions of both temperature and local composition. Effective thermal conductivity and permeability are estimated as functions of particle conversion.

**Figure 6:**
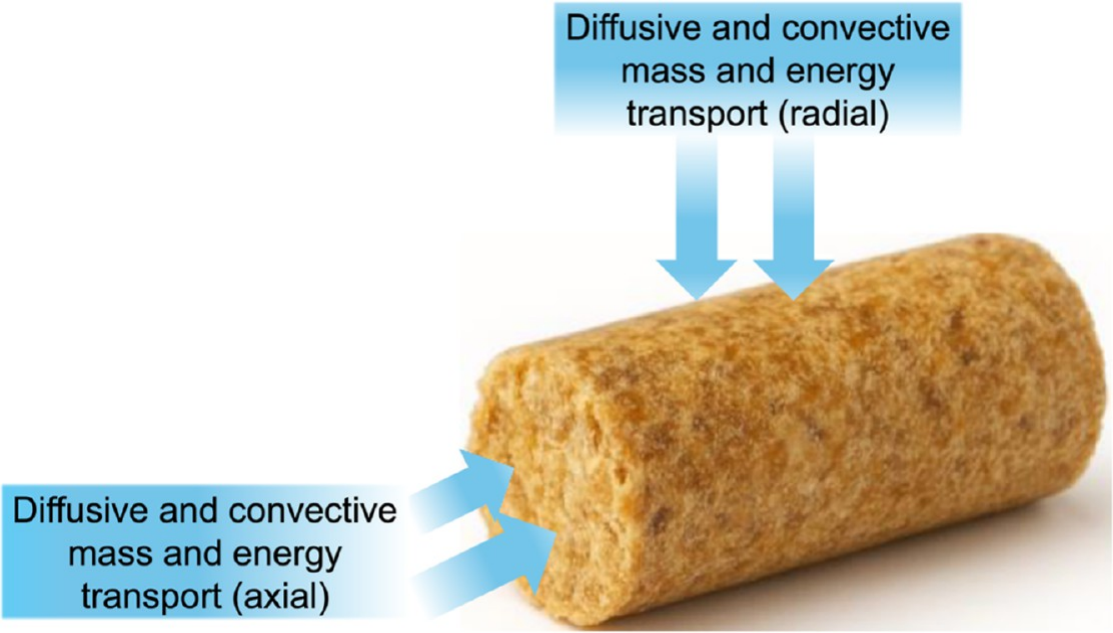
Illustration of the particle model used in this work.

##### 2.4.3.2 Reaction Scheme

The model employs the detailed kinetic framework introduced by Debiagi et al.^27^ as adapted by Sánchez et al.^48^ encompassing biomass pseudo-components and their liquid intermediates. In total, fifteen species are tracked: seven metaplastic intermediates (CELLA, HCE1, HCE2, LIG-CC, LIG-OH, and LIG), alongside three lignin fractions, consisting of hydrogen-rich lignin (LIG-H), oxygen-rich lignin (LIG-O), and carbon-rich lignin (LIG-C). Metaplastics comprises the oligomeric derivatives of cellulose, hemicellulose, and lignin, corresponding to the active intermediates in Debiagi’s scheme. In this work, softwood hemicellulose (GMSW) was considered to model the composition of pine feedstocks.

To maintain computational efficiency while accurately capturing particle mass-loss kinetics and product yields, all permanent gases (CO, CO_2_, CH_4_, and H_2_, plus light hydrocarbons such as ethene and propene) are lumped into a single gaseous component, and all volatiles—including low-molecular-weight cellulose species (carbonyls, hydroxyacetaldehyde, and sugar monomers/oligomers) and lignin-derived species (carbonyls, alcohols, and phenolics)—are similarly aggregated.^49^ The model also includes moisture evaporation and intraparticle vapor transport, and it integrates secondary intraparticle cracking and charring reactions of the volatiles following.^34^ Detailed kinetic parameters and reaction pathways are provided in Sánchez et al.^48^

##### 2.4.3.3 Mass Balances

In the pellet-scale formulation, both the solid and metaplastic (liquid) phases are treated as static in the mass-balance equations. Within the gas phase, species transport is described by both diffusive and convective fluxes in the radial and axial directions. The mass balances for the lumped permanent gases, the volatile (light condensable) fraction, and water vapor are expressed in eq 29. Here, the gas-phase porosity is defined as ε_G_ = 1 – ε_S_ – ε_L_, where ε_S_ and ε_L_ denote the local volume fractions of the solid and liquid phases, respectively. In the convective term, *v*_g_ represents the mixture velocity, while ρ_i_^*^ denotes the species mass concentration (per unit particle volume) for each of the gas-phase components. The corresponding source terms account for the in situ generation of permanent gases, volatiles, or water vapor, as dictated by the reaction mechanism. It is important to highlight that the strategy adopted involves expressing density in terms of the mass of the given species or the total gases per unit volume of the particle or pellet. In this sense, the particle porosity is implicitly included in the equations and is, therefore, not explicitly represented. However, during the software execution, the variation in volume (due to conversion, (η) evolves with time), and consequently, the variation in porosity is accounted for by quantifying the reduction in the volume of the integration domain.

29 $$\frac{\partial \rho_{i}^{*}}{\partial t}=\frac{\partial }{\partial x}\left(\frac{D_{{eff}_{G_{x}}}}{\varepsilon_{G}}\frac{\partial \rho_{i}^{*}}{\partial x}\right)+\frac{1}{r}\frac{\partial }{\partial_{r}}\left(r\frac{D_{{eff}_{G_{r}}}}{\varepsilon_{G}}\frac{\partial \rho_{i}^{*}}{\partial r}\right)-\frac{\partial \left(v_{G_{x}}\rho_{i}^{*}\right)}{\partial x}-\frac{\partial \left(v_{G_{r}}\rho_{i}^{*}\right)}{\partial r}+\sum_{j=1}^{N_{r}}{ṙ}_{i,j}^{\prime \prime \prime }$$

The estimation of gas mixture velocity—including permanent gases, volatile compounds, and water vapor—is based on Darcy’s Law (see eq 8), considering that bulk transport is mainly induced by intraparticle pressure gradients, while the internal flow of gaseous species is predominantly controlled by viscous forces.^44^ Permeability was calculated from Mesoflow simulations performed on the XCT geometries of cut pine, pelletized pine, and their corresponding chars and used in the particle models to calculate the gas mixture velocity. The pressure of the gas mixture, *P*, is calculated using the ideal gas law, based on the gaseous mixture’s density and average molecular weight.

#### 2.4.4 Energy Balance

The model considers diffusive and convective transport of energy in both the radial and axial direction, as well as the anisotropy ratio of the transport properties, i.e., thermal conductivity and permeability (see eq 30). Particle density is defined as ρ = ρ_L_^*^ + ρ_s_^*^ + ρ_g_^*^, where ρ_L_^*^,ρ_s_^*^, and ρ_g_^*^ are defined as the ratio between the mass of a specific component (liquid, solid, and gas, respectively) and the total particle volume. Advective heat transport arises from the motion of the gas-phase components—namely, permanent gases, volatiles, and water vapor. λ_effx_ and λ_effr_ correspond, respectively, to the axial and radial effective thermal conductivities of the pellet. The local heat capacity *C*_p_ is calculated as a function of temperature and considers the local fractions of the solid, liquid, and gaseous phases. ρ_i_^*^ and *C*_p,*i*_ correspond to the concentrations and heat capacities of each component within the gas phase. The source term corresponds to the sum of the enthalpies of the reactions of the *j* reactions associated with each species within the reaction mechanism. Reaction enthalpies are adopted from Debiagi et al.^27^

30 $$\begin{array}{l} \rho C_{p}\frac{\partial T}{\partial t}=\frac{\partial }{\partial x}\left(\lambda_{{eff}_{x}}\frac{\partial T}{\partial x}\right)+\frac{1}{r}\frac{\partial }{\partial_{r}}\left(r\lambda_{{eff}_{r}}\frac{\partial T}{\partial r}\right)-\frac{\partial \left(v_{G_{x}}\rho_{G_{i}}^{*}C_{p,i}T\right)}{\partial x}-\frac{\partial \left(v_{G_{r}}\rho_{G_{i}}^{*}C_{p,i}T\right)}{\partial r}\\ +\sum {ṙ}_{j,i}^{\prime \prime \prime }{\Delta H}_{R_{j}} \end{array}$$

##### 2.4.4.1 Boundary Conditions and Model Implementation

As initial conditions, uniform fields of pressure and temperature are considered. The solid matrix is initially composed of virgin biomass with initial porosity ε_g,0_, calculated from XCT geometries. Boundary conditions include convective heat and mass transfer at the pellet’s top surface and lateral walls.

The governing mass- and energy-balance equations are solved using a finite-difference scheme with an upwind stencil for the convective terms. Temporal integration is performed with MATLAB 2023a′s variable-step, stiff DAE solver (ode15s) to ensure numerical stability. At every time step, local thermophysical properties are updated from the nodewise temperature and species concentrations.

With respect to numerical accuracy, mesh-independent analyses targeting particle mass-loss rate and char yield revealed that solution quality is highly sensitive to the integration step; consequently, the maximum time step was restricted to 0.01 s. All simulations were executed on a Dell Precision 3551 mobile workstation equipped with an Intel Core i7-10850H CPU (2.70 GHz) and 32 GB RAM.

##### 2.4.4.2 Thermal and Transport Properties

Accurately simulating biomass pyrolysis at the particle scale hinges on specifying reliable transport parameters, yet the literature reports a wide range of values—and underlying assumptions—for highly sensitive constants such as thermal conductivity, mass permeability, and anisotropy ratios. Effective axial and radial conductivities are estimated according to eqs 31–33 based on the conversion, η, derived using the density of biomass species at time t divided by the initial biomass density, local fractions of biomass, char, and the local porosity, where λ_b_ and λ_c_ correspond to the temperature-dependent cell wall thermal conductivities of biomass and char.

31 $$\eta =1-\frac{\rho_{bm}}{\rho_{bm,0}}$$

32 $$\lambda_{s_{x,r}}=\left(1-\eta \right)\lambda_{b_{x,r}}+\eta \lambda_{c_{x,r}}$$

33 $$\lambda_{{eff}_{x,r}}=\varepsilon_{s}\lambda_{s_{x,r}}{+\varepsilon }_{G}\left(\lambda_{G}\right)$$

Additionally, the shrinkage parameters of the particle were used to update the particle dimensions as a function of the conversion. The shrinkage in the axial direction was calculated from measuring the lengths of cut pine and pelletized pine samples and chars according to eq 34, where *L*_f_ is the length of the char sample and *L*_0_ is the length of the native sample before pyrolysis:

34 $$s_{x}=\frac{L_{0}-L_{f}}{L_{0}}$$

similarly, the shrinkage of the particle in the radial direction was calculated from measurements of sample radii before and after pyrolysis according to eq 35, where *r*_f_ is the radius of the char sample and *r*_0_ is the radius of the native sample before pyrolysis.

35 $$s_{r}=\frac{r_{0}-r_{f}}{r_{0}}$$

Permeability was also calculated as a function of conversion, interpolating between the permeability of the virgin particle and the permeability of the pyrolyzed particle from Mesoflow. The physical and transport properties of the main components, such as the densities and thermal conductivities of the biomass and char solid wall, are shown in Table 2.

**Table 2:** Physical Properties Used in the Particle-Scale Model^a^

parameter	value	units
biomass cell wall density	1500	kg·m^–3^
char cell wall density	1700	kg·m^–3^
density of metaplastic phase, ρ_L_	1100	kg·m^–3^
specific heat capacity of biomass, *C*_p_b__	1500 + *T*	J·kg^–1^·K^–1^
specific heat capacity of char, *C*_pchar_	420 + 2.09 × *T* – 6.85 × 10^–4^ × *T* ^2^	J·kg^–1^·K^–1^
specific heat capacity of trapped moisture, *C*_p_acqua__	4200	J·kg^–1^·K^–1^
specific heat capacity of metaplastics, *C*_p_L__	2500	[J·kg^–1^·K^–1^]
specific heat capacity of water vapor, ${Cp}_{H_{2}O}$	1761 + 0.2316 × *T* + 0.0003 × *T* ^2^	[J·kg^–1^·K^–1^]
specific heat capacity of volatiles, *C*_p_v__	–100 + 4.4 × *T* – 1.57 × 10^–3^ × *T* ^2^	[J·kg^–1^·K^–1^]
specific heat capacity of light gases, *C*_p_g__	770 + 0.629*T* + 1.91 × 10–4 × *T*2	[J·kg^–1^·K^–1^]
biomass cell wall thermal conductivity, λ_b_	0.0159 + 7.36 × 10^–4^ × *T*	W·m^–1^·K^–1^
char cell wall thermal conductivity, λ_c_	1.47 + 0.0011 × *T*	W·m^–1^·K^–1^
thermal conductivity of metaplastics, λ_L_	0.5	W·m^–1^·K^–1^
thermal conductivity of gas mixture, λ_G_	λ_G_ = −0.0543 + 0.0003 × *T*	W·m^–1^·K^–1^
effective diffusion coefficient of gas mixture, $D_{eff\text{\_}G}$	3 × 10^–5^	m^2^·s^–2^
viscosity of gas mixture, μ_G_	3.85 × 10^–5^	Pa·s

a Additional nomenclature definitions are listed in the Supporting Information (Table S3).

Different cases were simulated using the particle model to investigate the impact of feedstock preprocessing and anisotropic transport, as follows:

- Case 1a: Pelletized pine particle model with both axial and radial mass transport and nearly isotropic thermal conductivity, where the radial thermal conductivity is slightly less than the axial thermal conductivity (λ_eff,r_ = 0.906λ_eff,x_)
- Case 1b: Cut pine particle model with both axial and radial mass transport and anisotropic thermal conductivity (λ_eff,r_ = 0.714λ_eff,x_) based on ref 48
- Case 2: Pelletized pine particle model based on case 1a but with no radial mass transport
- Case 3: Pelletized pine particle model based on case 1a but with high anisotropy in thermal conductivity (λ_eff,r_ = 0.580λ_eff,x_)

### 2.5 Experimental and Modeling Integration Strategy

This study combined information from SPR experiments, XCT imaging, reactor-scale modeling, and particle-scale modeling to elucidate the impact of feedstock preprocessing techniques on the conversion behavior of pine samples during pyrolysis. The approach to experimental and modeling integration utilized in this work is summarized in Table 3.

**Table 3:** Experimental and Modeling Integration Strategy

method	purpose	inputs	outputs
single-particle reactor (SPR) experiments	pyrolysis of pelletized or cut pine with different particle sizes and reactor temperatures, measurement of weight loss profiles; model validation	pelletized and cut pine samples of different sizes and different pyrolysis temperatures	temperature measurements from thermocouple 5 mm below the sample, sample weight loss measurements
X-ray computed tomography	microstructural imaging	representative samples of cut pine, pelletized pine, and chars after pyrolysis	3D geometries of internal microstructure of samples
MATBOX	analysis of XCT data	3D XCT geometries segmented into void and solid phases	calculated porosities, tortuosity factors, and Bruggeman exponents for cut and pelletized pine samples and chars
Mesoflow	permeability calculation	3D XCT geometries segmented into void and solid phases	principal permeabilities for cut and pelletized pine samples and chars
reactor-scale model	validation of experimental data and boundary condition identification for the particle model	density, permeability, and porosity from measurements and microstructural analysis	external temperature for convective heat transfer boundary condition in particle models, validation of heating rate
particle-scale model	detailed investigation of relevant intraparticle transport phenomena, a comparison of weight loss profiles to experimental observations	sample dimensions, density, permeability, and porosity from measurements and microstructural characterization, pyrolysis kinetic mechanism	weight loss profiles of samples, temperature and pressure gradients within samples

## 3 Results and Discussion

### 3.1 Microstructural Characterization

#### 3.1.1 X-ray Computed Tomography Imaging

One cubic millimeter (1 mm^3^) sub-volumes cropped from 3D reconstructions of the microstructures of cut pine, cut pine char, pelletized pine, and pelletized pine char collected using XCT are shown in Figures 7 and 8. 2D cross-sections in the center of the sub-volumes in each of the principal directions of all samples are shown in Figures 9 and 10. Striking differences between the microstructures of cut pine and pelletized pine can be observed visually from the XCT reconstructions. The anisotropic anatomical features of cut pine comprise a pore structure of array-like axial tracheids, radial ray cells, and tangential pits, whereas pelletized pine exhibited a pore structure characterized by isotropic fine cracks. The results from the XCT imaging reveal the extent to which the pelletization process nearly eliminated the anatomical features observed in the cut pine samples.

**Figure 7:**
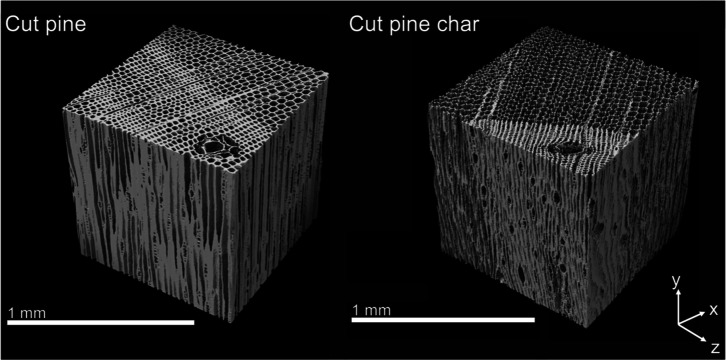
One cubic millimeter sub-volumes of cut pine and cut pine char XCT reconstructions used for microstructural analysis and permeability simulations.

**Figure 8:**
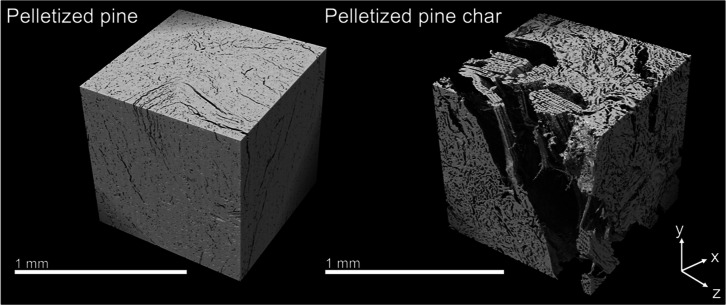
One cubic millimeter sub-volumes of pelletized pine and pelletized pine char XCT reconstructions used for microstructural analysis and permeability simulations.

**Figure 9:**
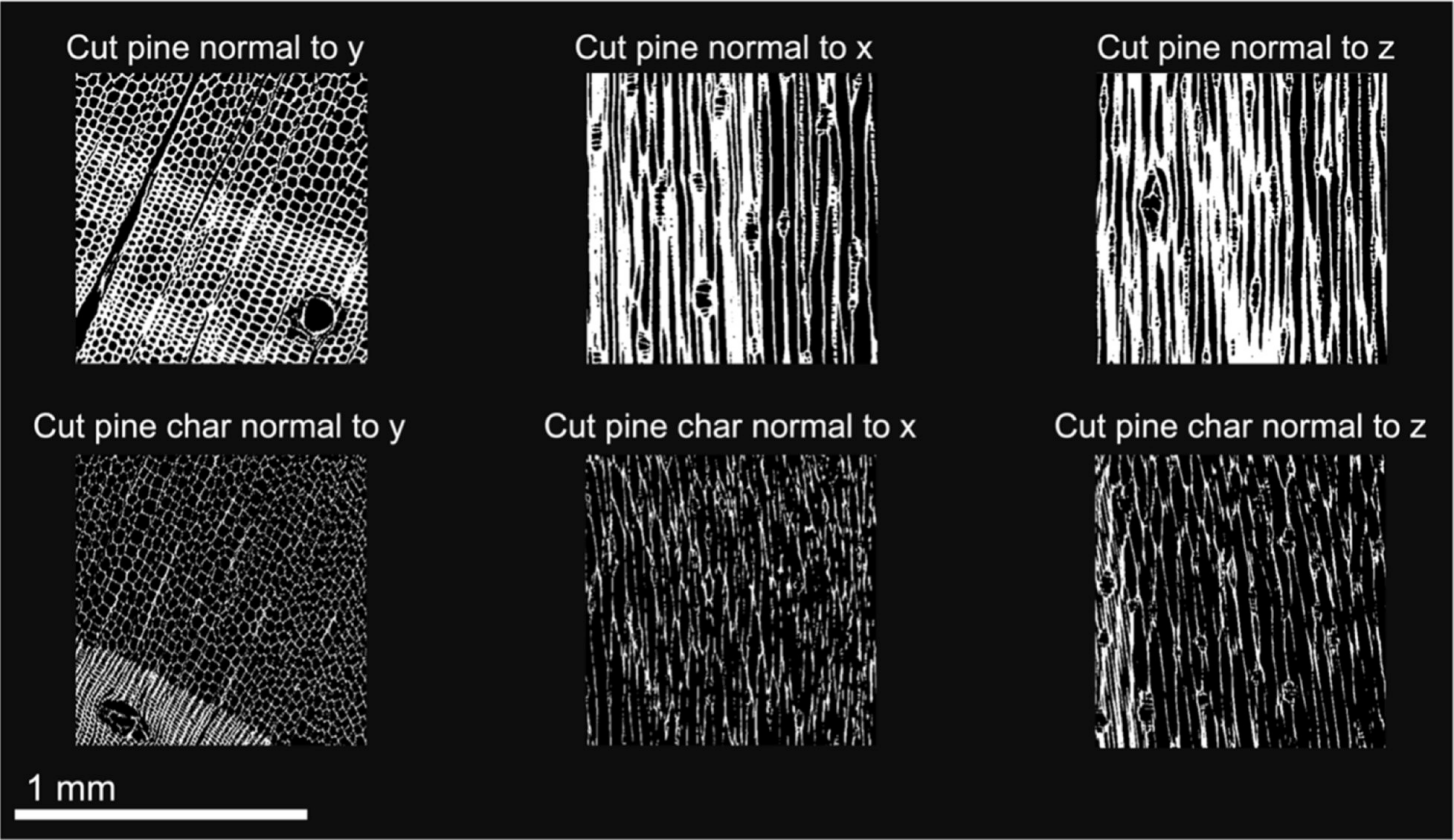
2D slices of XCT 1 mm^3^ sub-volumes normal to principal directions for cut pine and cut pine char samples.

**Figure 10:**
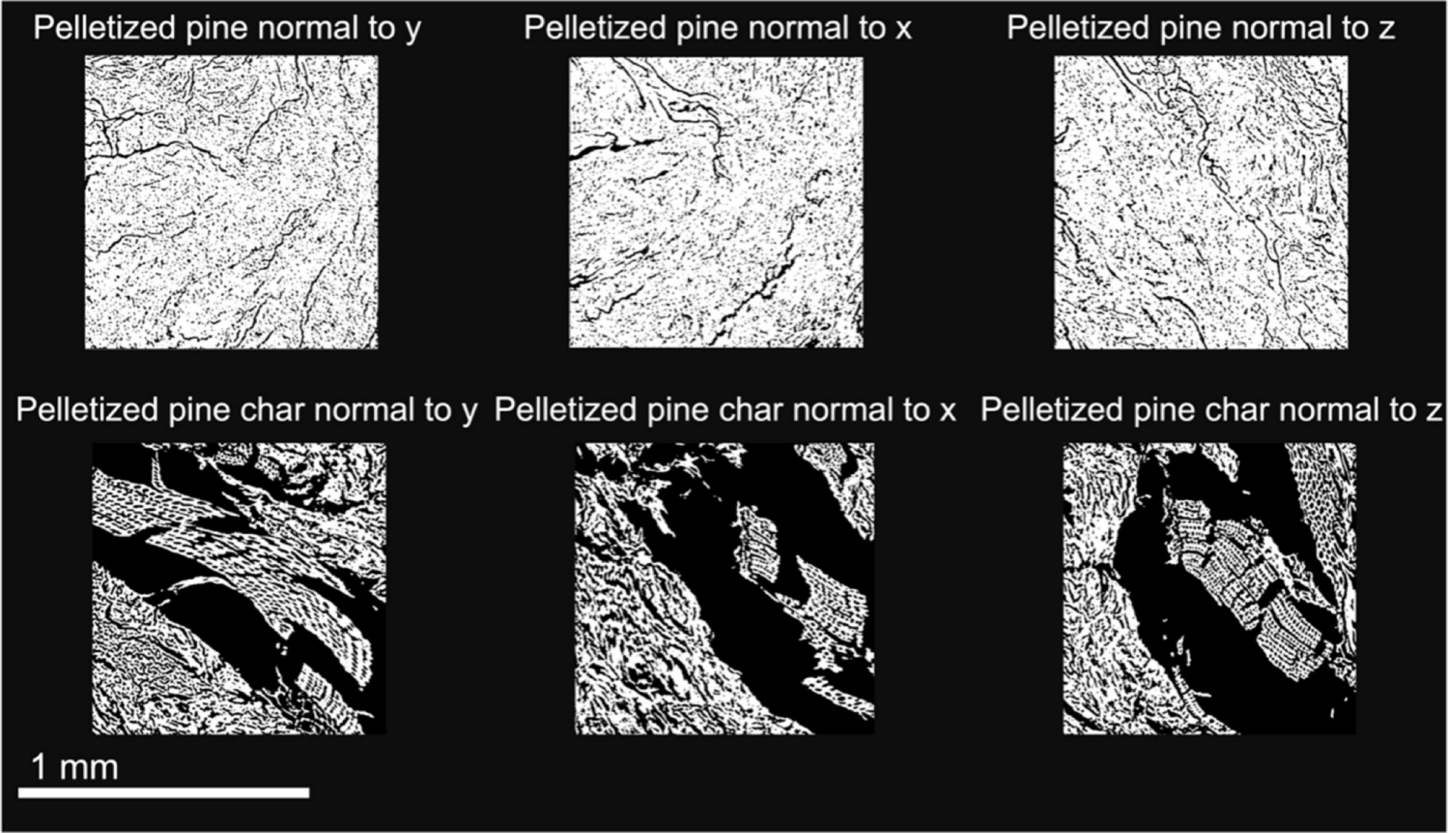
2D slices of XCT 1 mm^3^ sub-volumes normal to principal directions for pelletized pine and pelletized pine char samples.

The resultant chars after pyrolysis also exhibited significant differences in the microstructure. The cut pine char preserved much of the anatomical features, with pore degradation predominant in the radial and tangential directions. In contrast, the pelletized pine char microstructure was characterized by large voids potentially formed by the rapid expansion of volatiles within the material during pyrolysis. Additionally, the microstructure of the pelletized pine char was more isotropic than that of the cut pine char.

#### 3.1.2 MATBOX Calculations

The porosity, tortuosity factors, and Bruggeman exponents of cut pine and pelletized pine particles and chars are reported in Table 4. The trends in porosity revealed that residual chars after pyrolysis were more porous than the native pine material, both in the case of cut pine and pelletized pine, which was due to thinning of cell wall material, conjoining of some adjacent pores, and delamination of subparticles in the case of the pelletized material. Previous studies of biomass pyrolysis have also observed increased porosity after pyrolysis, and this phenomenon is generally thought to be due to the elimination of volatiles and accrual of pressure in the pores, condensation of amorphous carbon into more crystalline carbon, and degradation of cellulose, hemicellulose, and lignin in the plant cell wall.^6,50–53^

**Table 4:** Porosity, Tortuosity Factors, and Bruggeman Exponents of Cut Pine or Pelletized Pine Particles and Chars (Dimensionless)

sample	porosity	tortuosity factor, *x*	Bruggeman exponent, *x*	tortuosity factor, *y*	Bruggeman exponent, *y*	tortuosity factor, *z*	Bruggeman exponent, *z*
cut pine	0.57	24.64	6.77	1.15	1.26	54.45	8.20
cut pine char	0.82	1.98	4.46	1.07	1.33	1.98	4.46
pelletized pine	0.16	62.60	3.22	20.96	2.64	52.76	3.13
pelletized pine char	0.63	2.43	2.92	1.89	2.38	1.73	2.18

The anisotropy in the pore structure owing to anatomical features of pine wood was reflected in the values for tortuosity for cut pine, where the axial (*y*) direction had the lowest tortuosity and the radial (*x*) and tangential (*z*) directions had much higher tortuosity. The low tortuosity in the axial (*y*) direction can be explained by the presence of axial tracheid pores: long, predominantly straight pores aligned to the axial direction of the tree that are used to facilitate water and nutrient transport via capillary action. In the radial and tangential directions, ray cells and pits, respectively, facilitate transport from heartwood to bark and between adjacent cells, albeit more restricted than transport in the axial direction. This resulted in a higher calculated tortuosity in the radial (*x*) and tangential (*z*) directions for the cut pine sample. After pyrolysis, pore degradation resulted in anisotropy in cut pine char that was greatly reduced but still present. This was consistent with visual evidence of the native pore structure remaining in the cut pine XCT images.

In contrast to cut pine, the tortuosity in pelletized pine was very high in all directions, with the *y*-direction being the lowest. Since the pelletized material is an agglomeration of many smaller subparticles, one can no longer attribute the principal directions to the axial, tangential, or radial directions of the native pine material.

The pelletized pine char sample after pyrolysis had highly isotropic tortuosity, which was much lower than the pelletized pine before pyrolysis. This stark decrease in tortuosity and anisotropy of the pelletized pine microstructure was a result of the expansion of large voids in the pelletized pine char. These large voids visible in the XCT data facilitated direct, largely isotropic transport throughout the pelletized pine char, in contrast to the fine crack-like pores in the pelletized pine particle and the anisotropic pore structure of the cut pine particle and char.

The Bruggeman exponents for pine and pine char were significantly different between their cut and pelletized states. This indicates that the pelletization process not only induced a decrease in porosity but also drastically changed the solid domain morphology. This quantifies the visually noticeable differences visible between Figures 9 and 10. Along the *x* and *z* directions, the Bruggeman exponent was systematically lower for the pelletized samples. This indicates that the decrease in effective diffusivity was due to the porosity collapse but not to the particle shape change. On the contrary, along the *y* axis, the Bruggeman exponent was higher for the pelletized samples, indicating that the decrease in effective diffusivity was due to both a reduction in porosity and a change of particle morphology.

The calculated porosities for each sample were used in particle-scale models to define the void fraction within the particle: the initial value for ε_G_ for cut and pelletized pine. This allowed for the accurate calculation of energy, mass, and momentum transport for the cut and pelletized pine. In reactor-scale models, the porosities of the cut and pelletized pine samples were also used for ε_G_.

#### 3.1.3 Mesoflow Simulations

The diagonal components of the permeability tensors for cut pine and pelletized pine before and after pyrolysis were calculated from feature-resolved porous flow simulations through the 3D particle geometries obtained by using XCT. These simulations were performed using Mesoflow. Steady-state visualization of the velocity field through the particle microstructures is shown in Figure 11. The calculated permeabilities for each sample are reported in Table 5.

**Figure 11:**
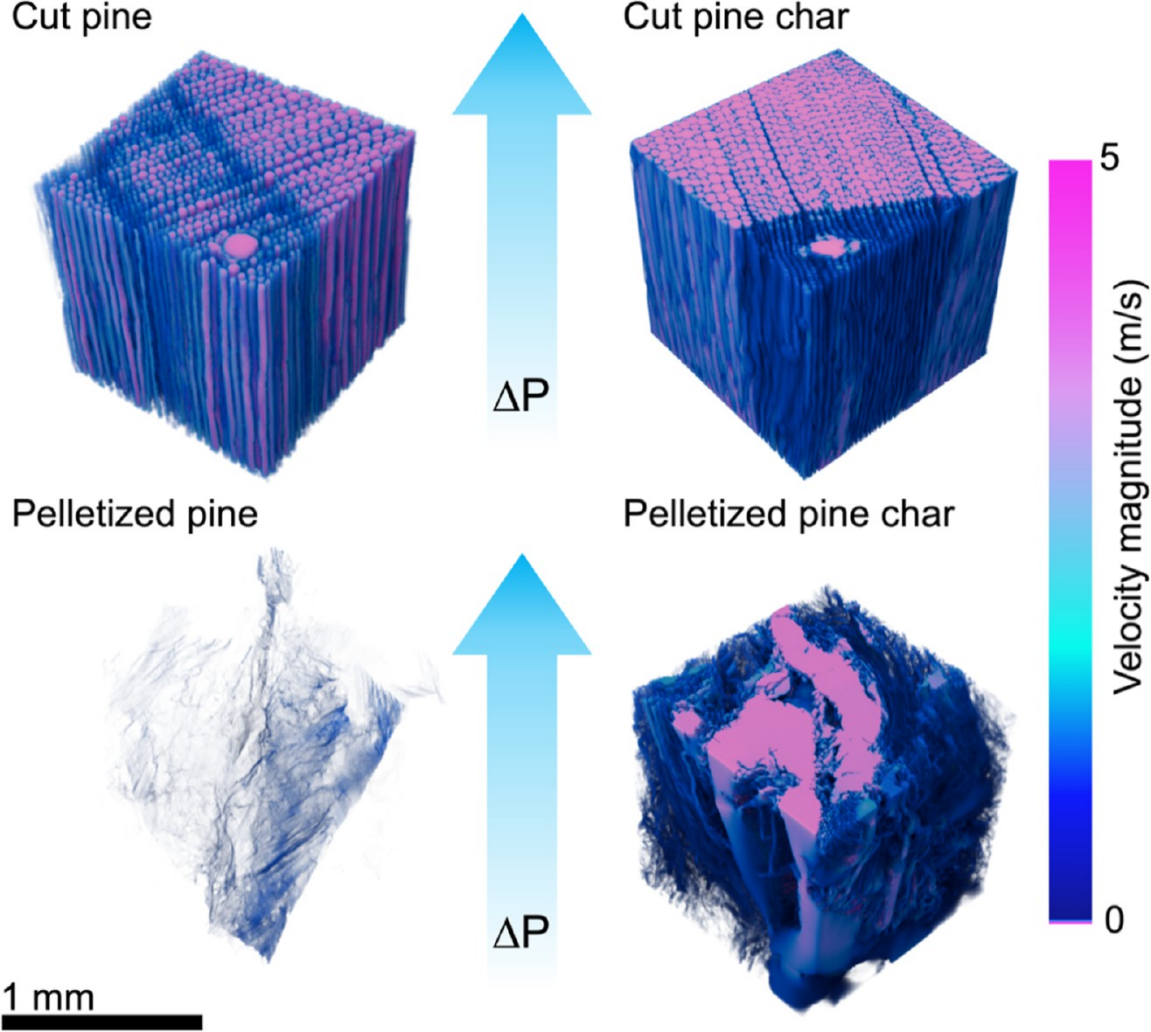
Velocity magnitude of fluid through the microstructure of cut pine and pelletized pine and chars at steady state from Mesoflow permeability simulations. Significant axial flow can be seen in the cut pine, cut pine char, and pelletized pine char samples; however, the pelletized pine was too dense to allow for significant transport through pores.

**Table 5:** Permeabilities in Each Direction of Cut Pine or Pelletized Pine Particles and Chars in m^2^

sample	*x*	*y*	*z*
cut pine	3.06 × 10^–13^	2.44 × 10^–12^	1.12 × 10^–14^
cut pine char	6.15 × 10^–13^	4.98 × 10^–12^	6.68 × 10^–13^
pelletized pine	3.74 × 10^–16^	1.55 × 10^–15^	1.45 × 10^–16^
pelletized pine char	2.63 × 10^–12^	4.24 × 10^–12^	4.87 × 10^–12^

The trends in permeability largely agreed with the trends in the tortuosity for each sample. The pelletized pine sample was much less permeable than the cut pine sample, evidenced by lower permeabilities in all directions by 2–3 orders of magnitude compared to cut pine. This result was due to the high tortuosity and low porosity of the material, which inhibited gas flow through the microstructure. All three directions had low permeability for the pelletized pine sample, although the *y* direction had the highest permeability among the three directions.

The natural anisotropy of cut pine was reflected in the highest permeability in the axial (*y*) direction, the direction parallel to the axial tracheid pores that conveys air, water, and nutrient flow up the length of the trunk of the tree. The permeabilities for cut pine were lower in the radial and axial directions, consistent with higher tortuosity measurements. After pyrolysis, the anisotropy was reduced in native cut pine char. This was consistent with an increased porosity and reduced tortuosity after pyrolysis, allowing for more direct flow in all directions. The pelletized pine char also exhibited a significant increase in permeability in all three directions after pyrolysis, even more so than the cut pine. The narrow pores in the pelletized pine microstructure may build up pressure more easily than larger pores as pyrolysis vapors form and condense, resulting in the formation of larger pores during conversion that allow for facile transport in the char. The calculated permeability tensors for each sample were used in particle- and reactor-scale models for accurate, feedstock-specific momentum transport.

### 3.2 Single-Particle Experimental Results

The fraction of initial particle mass over time calculated from the measured weight of each sample during pyrolysis experiments in the SPR is shown in Figures 12–14. Figure 12 reveals the impact of feedstock preprocessing techniques (cutting vs pelletization) on the weight loss of pine samples of the same size. The results indicate that the cut pine samples lost mass more rapidly than the pelletized pine samples. Additionally, at steady state, more residual char remained in the pelletized pine sample compared to the cut pine sample of the same diameter. After 100 s of pyrolysis at 650 °C, the average of the 6 mm diameter pelletized particles had 19% residual mass, whereas the average of the cut pine samples had only 14%. This result was largely attributed to the increased thermal mass due to the high density of the pelletized pine particle, which kept the particle at lower temperatures over the course of pyrolysis. Pyrolysis at lower temperatures tends to favor char formation rather than volatile production.^34^ Less solid biomass, therefore, was volatilized into gaseous products, leading to more residual mass at a steady state.

**Figure 12:**
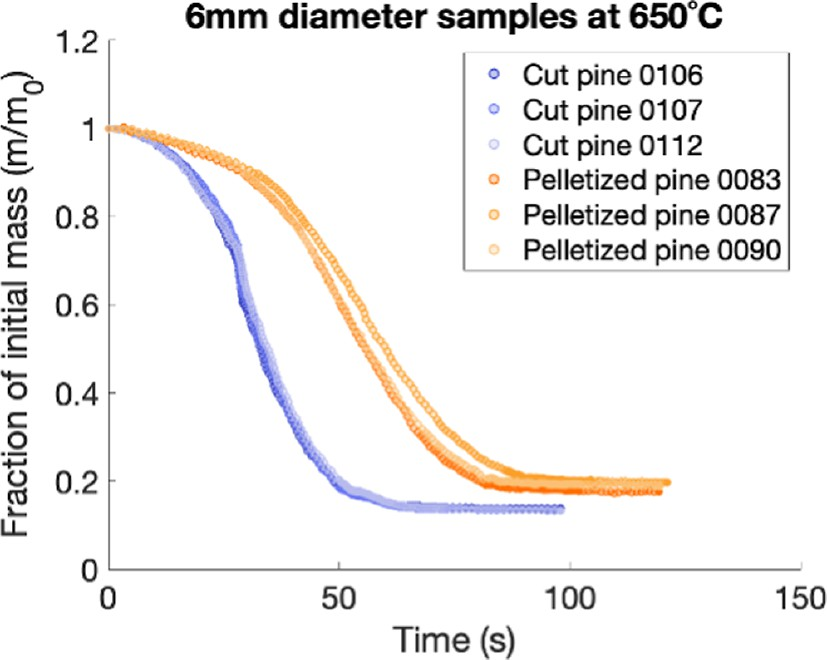
Impact of feedstock preprocessing on the mass loss profile over time of samples during pyrolysis at 650 °C in the SPR. Cut pine samples are represented by the blue data points and pelletized pine samples are represented by the orange datapoints. The numbers following each sample correspond to sample IDs on the Bioenergy DataHub.

Figure 13 shows the impact of sample size (increased diameter and length) on the mass loss during pyrolysis in the SPR for 6 and 9 mm cut pine samples. As expected, the smaller 6 mm diameter particle had more rapid mass loss than the larger 9 mm diameter particle, and the residual mass at steady state is also larger for the 9 mm diameter particle. Greater internal heat transfer resistance in the larger particles led to slower mass loss, which impeded thermally activated volatilization reactions, resulting in more residual char.

**Figure 13:**
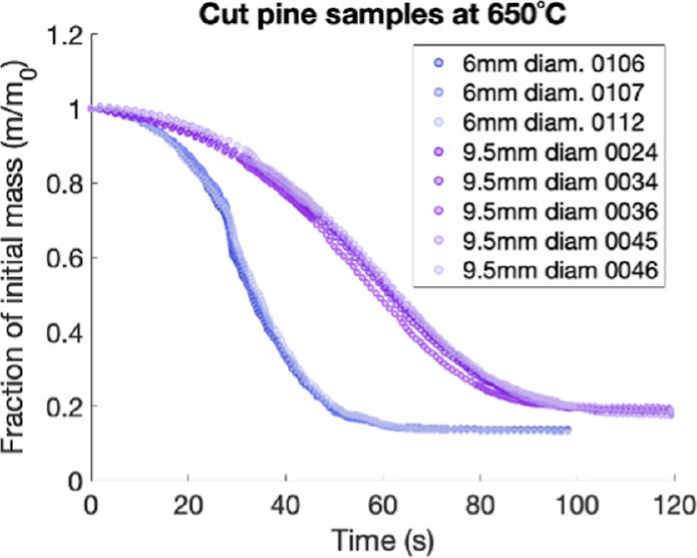
Impact of sample size on the mass loss profile over time of cut pine samples during pyrolysis at 650 °C in the SPR. 6 mm diameter cut pine samples are represented by blue datapoints and 9.5 mm diameter cut pine samples are represented by purple datapoints. The numbers following each sample correspond to sample IDs on the Bioenergy DataHub.

Figure 14 reveals the impact of different pyrolysis temperatures on the mass loss of cut and pelletized pine samples. The increased temperature increased the rate of mass loss for both cut pine and pelletized pine particles. Higher pyrolysis temperature led to more rapid sample heating, which promoted volatilization reactions and led to slightly less residual char in both sample types.

**Figure 14:**
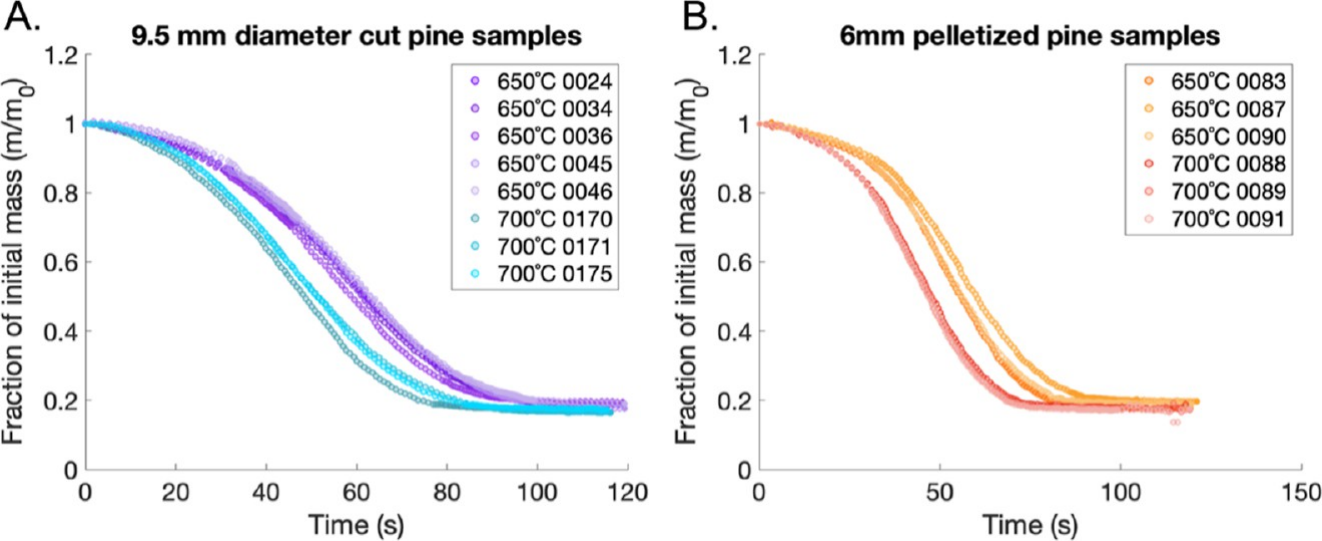
Impact of pyrolysis temperature on the mass loss profile over time during experiments in the SPR. (A) The fraction of initial sample mass over time for cut pine samples at two different temperatures is presented. 9.5 mm diameter cut pine samples pyrolyzed at 650 °C are represented by the purple data points, and 9.5 mm diameter cut pine samples pyrolyzed at 700 °C are represented by the cyan data points. (B) The fraction of initial sample mass over time for 6 mm diameter pelletized pine samples at two different temperatures is presented. 6 mm diameter pelletized pine samples pyrolyzed at 650 °C are represented by the orange data points, and 6 mm diameter pelletized pine samples pyrolyzed at 700 °C are represented by the red data points. The numbers following each sample correspond to sample IDs on DataHub.

The results from SPR experiments revealed the interplay of feedstock preprocessing, particle size, and pyrolysis temperature on pyrolysis conversion times and product distributions. The conversion time was fastest for 6 mm diameter cut pine samples, producing the least residual char due to facile transport through their porous, permeable microstructures. Pelletized pine samples converted more slowly due to their increased thermal mass. Larger particles also heated more slowly due to their decreased surface-area-to-volume ratios and produced more residual char. While higher temperatures led to faster conversion times and less residual char for both cut pine and pelletized pine samples, it has been observed that more gas phase cracking reactions occur at elevated temperatures, reducing the pyrolysis oil yield.^54^ Therefore, higher pyrolysis temperatures are typically suboptimal for industrial applications in which the pyrolysis oil is the desired product.

### 3.3 Reactor Modeling

A reactor model for the SPR was developed in COMSOL Multiphysics v6.1 to identify boundary conditions used in single-particle simulations.^23^ The measured density, porosity calculated in MATBOX, and permeability calculated from Mesoflow simulations were used to parametrize reactor models, including cut pine and pelletized pine samples. The recorded wall temperatures from SPR experiments were used as temperature boundary conditions at the reactor wall in the model, and the model-predicted temperature at the position of the thermocouple 5 mm below the sample, was compared to experimental measurements. Figure 15 shows the temperature profile in the reactor at 0.85 s and the velocity magnitude at 300 s.

**Figure 15:**
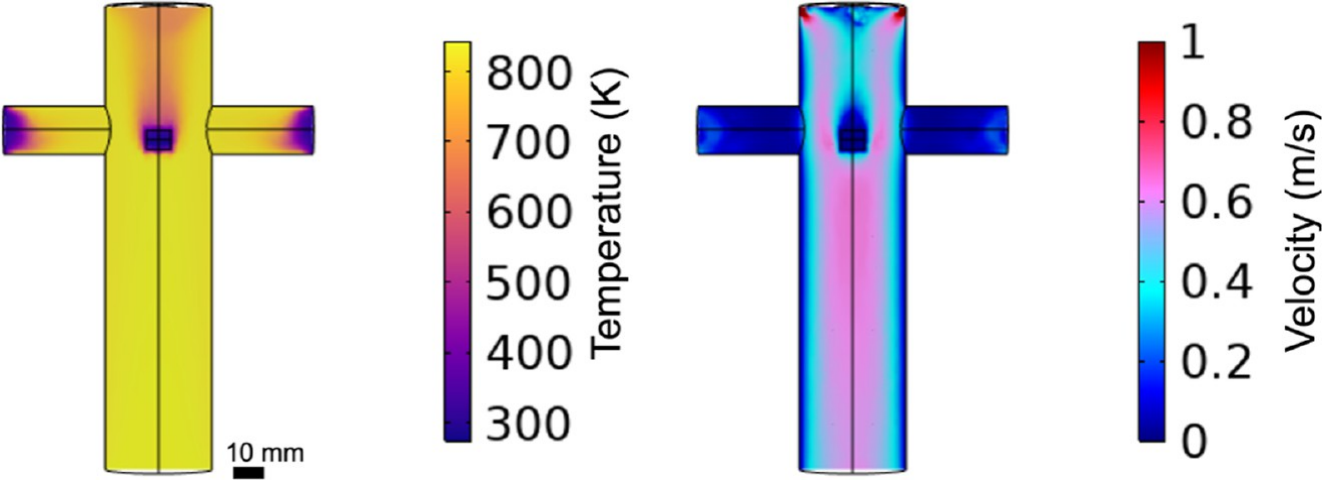
Temperature profiles of a slice through the center of the reactor at 0.85 s and velocity profile at 300 s of SPR model simulation with pine particle. The pelletized pine particle simulations are indistinguishable for the fluid domain.

Figure 16 shows the comparison of the reactor temperature predicted by reactor-scale models to experimental measurements for simulations of the SPR with a cut pine particle or a pelletized pine particle. The experimental data used for comparison was from thermocouple measurements of temperature during pyrolysis of a pine particle or a pelletized pine particle in the SPR. A point probe was defined in the model at the position of the thermocouple in SPR experiments, 5 mm below the bottom of the particle and 8 mm from the center of the reactor. In general, the model agreed well with experimental measurements for both the pine particle and pelletized pine particle. However, the model slightly overestimated the heating rate of the reactor at early times and slightly under-predicted the temperature at later times. This may be due to the material properties of the thermocouple, which could have delayed the measured temperature at early times due to heat transfer limitations and overestimated the later temperatures due to radiation. The reactor model confirms the rapid heating rate experienced by the samples in the SPR experiments, with over 100 K/s in the first few seconds.

**Figure 16:**
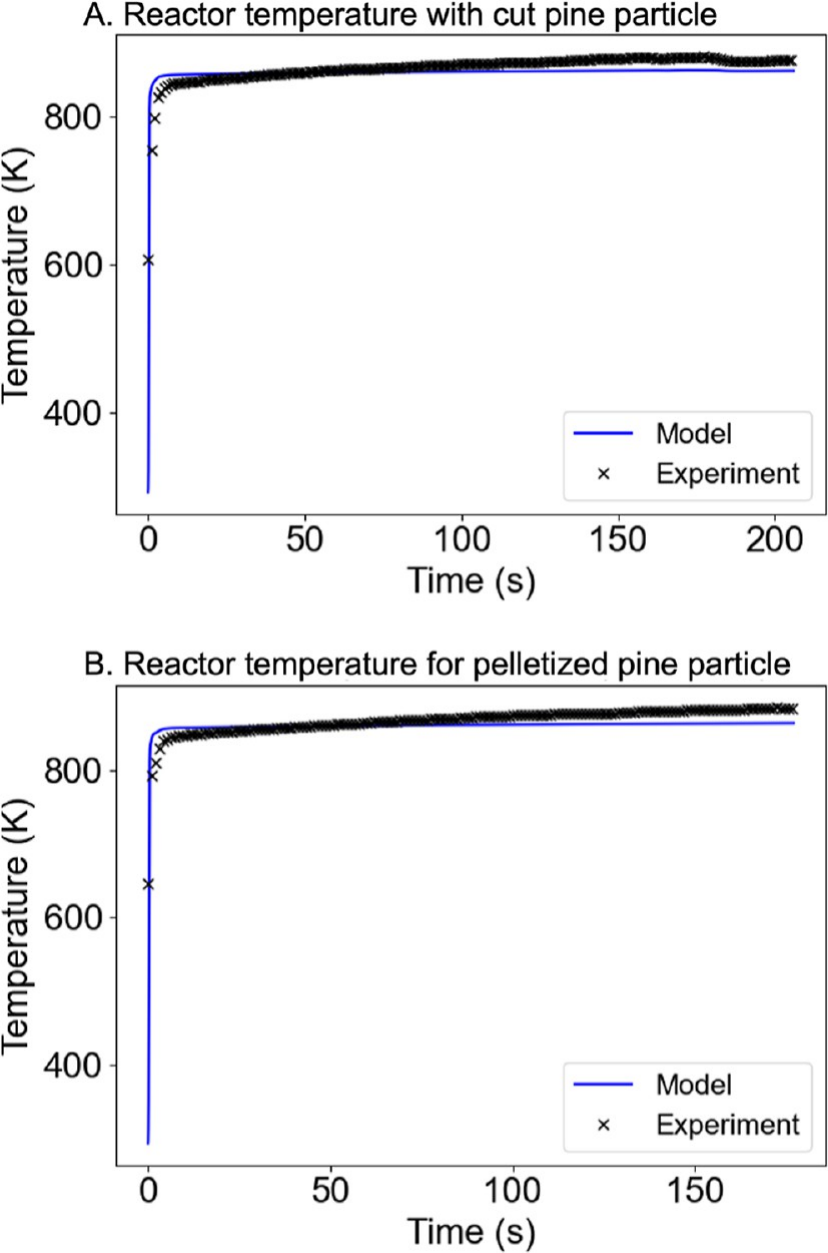
Comparison of model-predicted reactor temperature at a point probe at the location of the thermocouple 5 mm below the sample to experimental thermocouple measurements from SPR.

In addition, the volume averaged particle temperature over time was predicted by the reactor models for pelletized pine and cut pine, compared in Figure S1. This shows that the pelletized pine experienced a lower temperature and slower heating rate compared to the cut pine particle.

### 3.4 Single-Particle Pyrolysis Modeling and Comparison to Experimental Results

Single-particle models were developed in MATLAB 2023a to investigate the impact of intraparticle transport on the conversion behavior of cut or pelletized pine feedstocks during pyrolysis. Models were parametrized for cut pine and pelletized pine particles by including the calculated porosities of the materials before and after pyrolysis from MATBOX, geometric dimensions and density measured directly from the samples, and axial and radial permeabilities before and after pyrolysis calculated from simulations in Mesoflow. A convective heat transfer boundary condition was enforced at the edge of the particle with a heat transfer coefficient of 90 W·m^–2^ K^–1^ and the surrounding temperature taken from thermocouple measurements as obtained from the reactor experiments and simulation results presented in Figure 16.

The residual mass profile of 6 mm diameter cut pine and pelletized pine predicted by the particle-scale model compared to the experimental mean for each sample pyrolyzed at 650 °C is reported in Figure 17A shows the results from particle models using the CRECK mechanism as reported in ref 27, with additional secondary reactions following ref 34 to model biomass conversion during pyrolysis in both cut pine and pelletized pine models. The weight loss profiles revealed that differences in porosity and permeability of the cut pine and pelletized pine led to different conversion behaviors, with pelletized pine losing mass slower than cut pine. This observation was reproduced by the model, suggesting that the transport limitations induced by the higher density, lower porosity, and lower axial and radial permeabilities in pelletized pine were well captured by the model. However, the model overpredicted the final residual mass for cut pine. Notably, the cut pine model with unmodified kinetics agreed well with the weight loss before 40 s. However, the cut pine model deviated from experimental observations at later conversion times.

**Figure 17:**
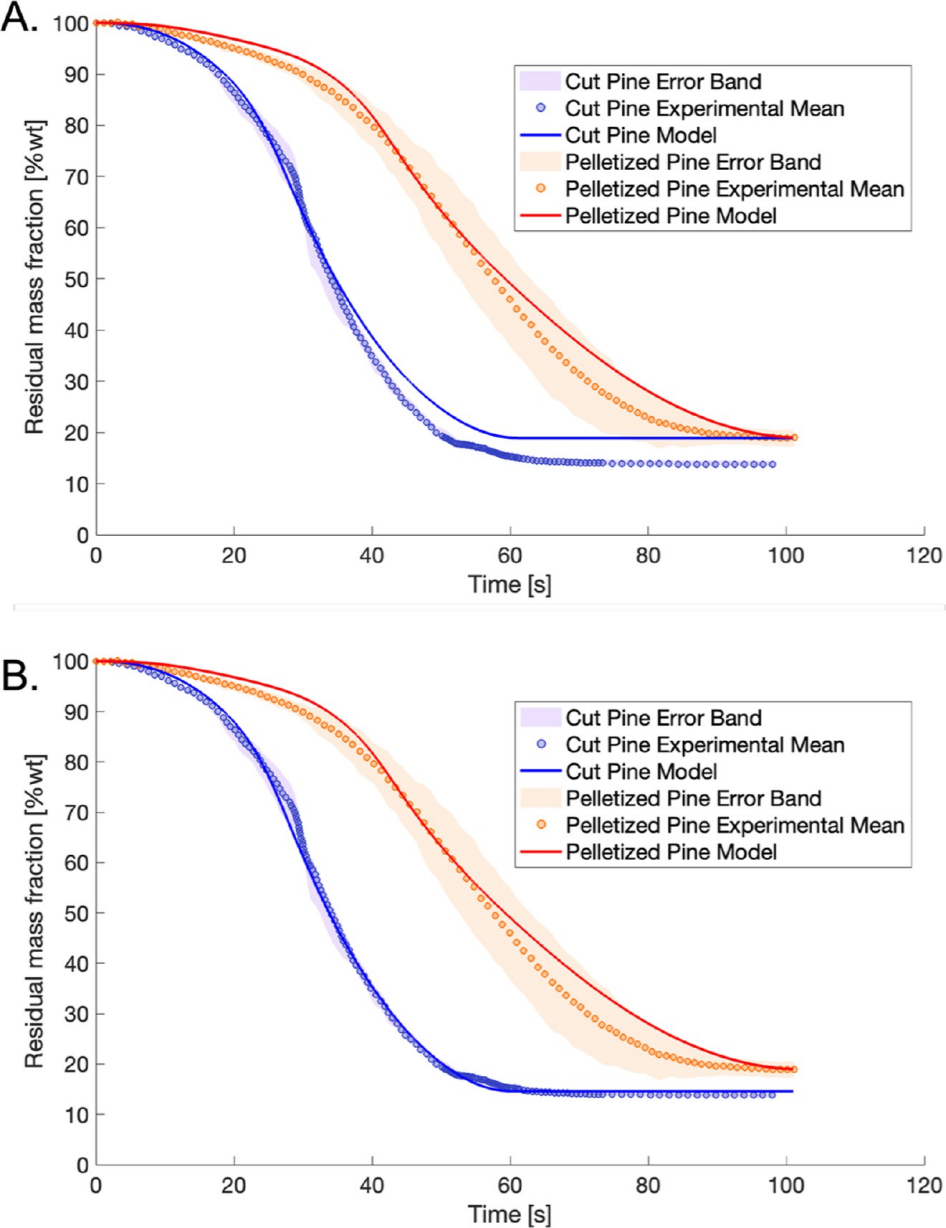
Comparison of 6 mm diameter cut pine and pelletized pine at 650 °C reactor temperature predicted by model (case 1a and case 1b) compared to experimental means with error bands. Model predictions are assuming: (A) original CRECK biomass pyrolysis kinetics according to ref 27 and secondary reactions from ref 34. (B) Modified kinetic parameters on char and metaplastic forming reactions as reported in Table S4 for cut pine only.

To address the overprediction of the final residual mass of cut pine by the particle model, the activation energies of several of the char and metaplastic forming reactions were increased from their original values. The residual solid mass after pyrolysis consists of char and metaplastic species; therefore, reactions involving their production were modified to better fit model predictions to experimental data. The model-predicted mass loss profile for cut pine shown in Figure 17B using the modified kinetics shows much better agreement to experimental observation. Previous studies have found that the models using the CRECK mechanism can over- or under-predict char yields depending on biomass feedstock characteristics and experimental conditions.^27,55–57^ In particular, the CRECK mechanism as presented in ref 27 performs well for low and intermediate heating rates around 30 K/min, yet the high heating rate of SPR experiments used in this study exceeded 100 K/s. Low heating rates are indicative of slow pyrolysis, which favors char production.^34^ The pelletized pine experienced a lower effective heating rate during pyrolysis due to its increased thermal mass, resulting in a residual mass profile in better agreement with the unmodified CRECK kinetics. These results suggest that the transport limitations were well captured by the model without unmodified kinetics; however, an increase in the activation energies of several char and metaplastic formation reactions provided a better fit to the experimentally observed char yield for cut pine when pyrolyzed at the relatively high heating rates achieved in the present study. The modified kinetic parameters for cut pine are reported in Table S4.

The pressures evaluated at the center of cut pine and pelletized pine particle models over time are shown in Figure 18. The pelletized pine sample exhibited nearly 10% higher pressure within the particle compared to cut pine, consistent with restricted transport through the low porosity, low permeability pore structure of the pelletized pine. The increase in pressure observed over time was due to the formation of volatile gases within pores, which once formed could not easily escape the pelletized pine. In contrast, the volatile products formed in the cut pine escaped more easily through the more porous and permeable microstructure, causing less pressure to build. High-pressure pyrolysis regimes have been shown to favor char-forming reactions,^58^ consistent with the higher residual mass observed in pelletized pine models (Figure 17). While the CRECK pyrolysis mechanism scheme used in this work does not explicitly incorporate pressure dependence on Arrhenius parameters, it was able to reproduce experimental observations of residual mass loss.

**Figure 18:**
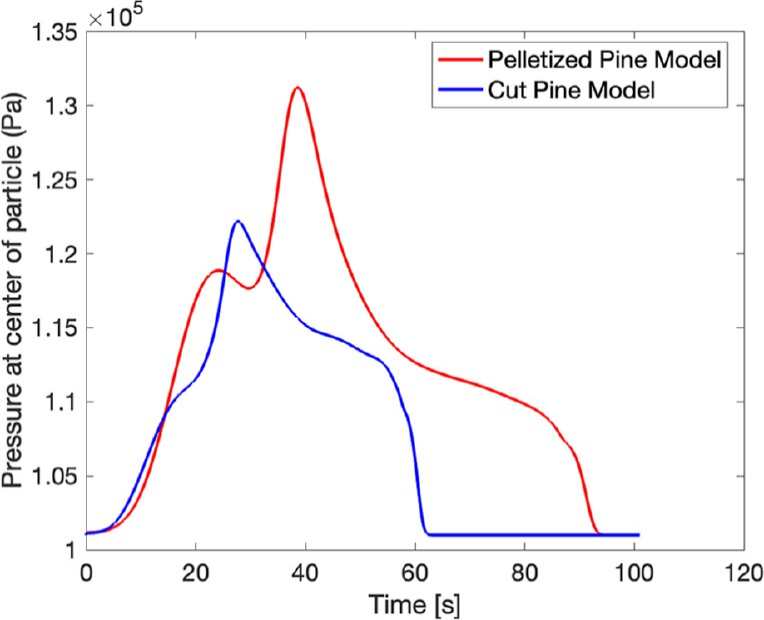
Comparison of the pressure at the center of 6 mm diameter cut pine (blue) and pelletized pine (red) over time at 650 °C reactor temperature predicted by particle models (case 1a and case 1b).

Dimensionless numbers, including the Biot number (*Bi*) and Pyrolysis numbers (Py and Py'), were used to characterize heat transfer regimes within cut pine and pelletized pine particle models. The Biot number probes the thermal resistance from the ratio of external convective and conductive heat transfer within the body, as given by eq 36.

36 $$Bi=\frac{h_{conv}r}{\lambda_{eff}}$$

where *h*_conv_ is the external convective heat transfer coefficient, *r* is the characteristic length (radius for radial direction and length for axial direction), and λ_eff_ is the effective thermal conductivity in the radial or axial direction, calculated using a sum of biomass, metaplastics, and char thermal conductivities weighted by their volume fraction in the particle over time:

37 $$\lambda_{eff}=\lambda_{b}\varepsilon_{b}\left(t\right)+\lambda_{c}\varepsilon_{c}\left(t\right)+\lambda_{L}\varepsilon_{L}\left(t\right)$$

Figure 19 shows the Biot number averaged over the particle in the axial and radial directions from the cut pine and pelletized pine particle models. In both cut pine and pelletized pine, the Biot number was higher in the axial direction than in the radial direction. This was due to the anisotropy in shape and thermal conductivity. For cut pine, the anisotropy in thermal conductivity was greater than in the pelletized pine and became nearly isotropic as more char formed. For the entirety of pyrolysis in both pelletized and cut pine particle models, the Biot number remained above one, suggesting the heat transfer during pyrolysis was limited by conduction rather than external convection. Consequently, a distinct temperature gradient developed within the particle, with the highest temperatures occurring near the external surface.

**Figure 19:**
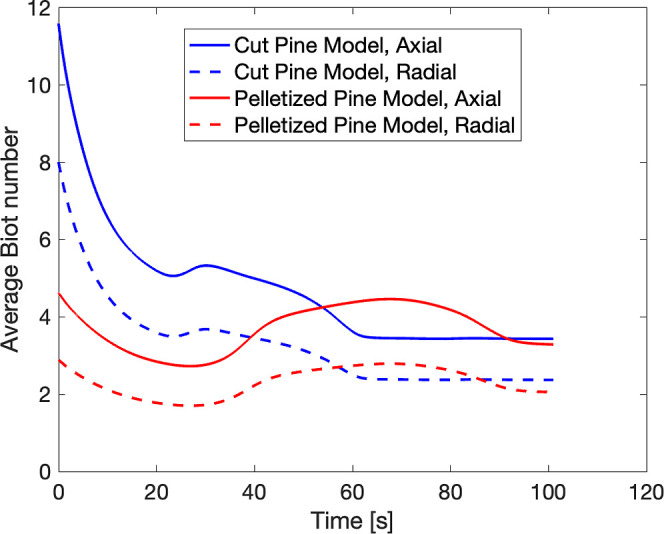
Comparison of the average Biot number of 6 mm diameter cut pine (blue) and pelletized pine (red) over time at 650 °C reactor temperature predicted by model (case 1a and case 1b).

The dimensionless pyrolysis numbers Py and Py′ as developed by Pyle and Zaror^59^ examine the relationship between heat transfer and the reaction rate of devolatilization during pyrolysis. The first pyrolysis number, Py, compares thermal conductivity to the devolatilization rate, given by:

38 $$Py=\frac{\lambda_{eff}}{k_{devol}\rho_{part}C_{p}r^{2}}$$

The second pyrolysis number, Py′, compares the external convective heat transfer coefficient to the devolatilization rate, given by:

39 $$Py^{\prime }=\frac{h_{conv}}{k_{devol}\rho_{part}C_{p}r}$$

where λ_eff_ is the effective thermal conductivity in the radial or axial direction, *h*_conv_ is the external convective heat transfer coefficient, ρ_part_ is the density of the particle, and *C*_p_ is the heat capacity of the particle. Here, the devolatilization reaction rate, *k*_devol_, is calculated using a finite-difference approach from the mass loss profile over time, according to:

$$k_{devol}=\frac{1}{m}\frac{dm}{dt}$$ 40

The log of the pyrolysis numbers averaged over the particle and evaluated over time for cut pine and pelletized pine models in the axial and radial directions is shown in Figure 20. Along the second *y*-axis, the devolatilization rate is shown. During pyrolysis, when the devolatilization rate is most negative, the order of magnitude of the pyrolysis numbers dropped near or below 0, indicative of the increasing role of heat transfer limitations (rather than reaction rate limitations) on pyrolysis. In general, the magnitude of log­(Py) was more negative than log­(Py′) for both cut pine and pelletized pine models, meaning conduction limitations were more prominent than external convection. Moreover, the pelletized pine had a more negative magnitude log­(Py) and log­(Py′) compared to the cut pine due to the higher density of the pelletized pine particle.

**Figure 20:**
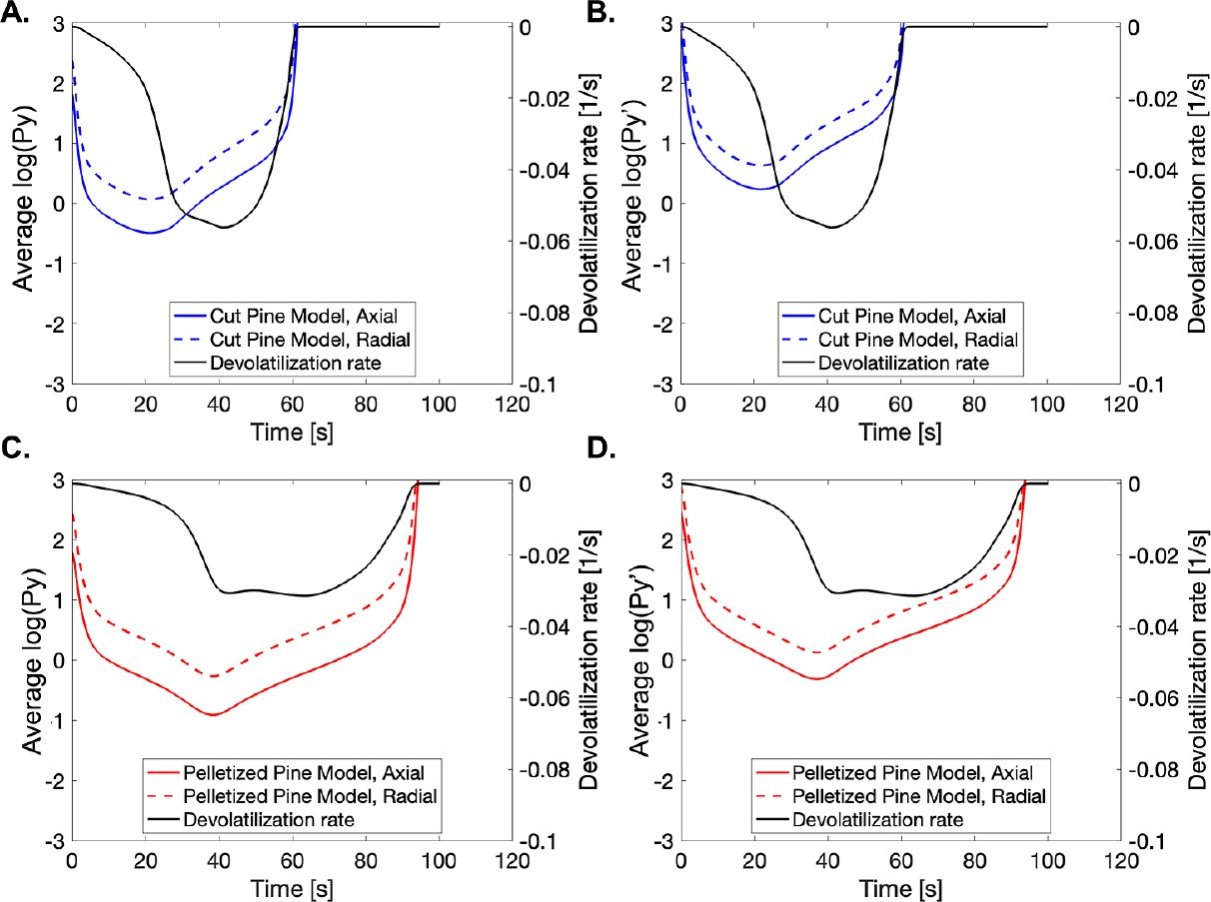
Average of the log of Pyrolysis numbers Py and Py′ over time for cut pine (blue) and pelletized pine (red) in axial (solid lines) and radial (dashed lines) directions. The devolatilization rate over time is plotted in black on the second *y*-axis.

Analysis of dimensionless numbers, including *Bi*, Py, and Py′ reveals the importance of capturing accurate anisotropic transport properties of biomass feedstocks. The combination of *Bi* on the order of 1E0 and both Py and Py′ on the order of 1 × 10^1^ to 1 × 10^–1^ during pyrolysis placed both the cut pine and pelletized pine in the competing heat transfer regime, where no single mode of heat transfer dominates entirely. Rather, the synergy of multiple heat transfer phenomena dictated the pyrolysis behavior. This result is consistent with findings from previous anisotropic particle models.^60^ Chiefly, the complexity of the heat transfer regime during the pyrolysis of biomass samples emphasizes the need for models that capture the microstructure-specific heat transport via conduction, convection, and chemical reactions to accurately predict the pyrolysis behavior.

Parametric sensitivity analysis was performed to evaluate how mass and energy transport properties affect the conversion of biomass at the single particle scale. For pelletized pine, the study considered effects of (I) radial mass transport (mass permeability anisotropy) and (II) thermal-conductivity anisotropy. Three representative cases were compared, covering the isotropic and highly anisotropic scenarios within these two categories. Table 6 lists the physical properties of the pelletized pine particles used in the analysis. The initial composition of cellulose (CELL), hemicellulose (GMSW), lignin (LIGC, LIGH, and LIGO), and moisture in the particle was determined using the experimental protocol described in Starace et al.^61^ The reactor temperature in particle models was taken from experimental measurements shown in Figure 16.

**Table 6:** Physical Properties Used in the Pelletized Pine Particle Model for Sensitivity Analysis Simulations^a^ ^,^

parameter	value	units
particle diameter	5.89	mm
particle length	8.12	mm
ρ_particle,0_	1239.24	kg·m^–3^
ε_g,0_	0.16	-
moisture content	0.061	(w/w)
*T*_gas,reactor_	from thermocouple measurements	°C
*T*_particle,*t*=0_	27	°C
*h*(combined)	90	W·m^–2^·K^–1^
CELL	0.410	(w/w), dry
HCE (GMSW)	0.245	(w/w), dry
LIGC	0.000	(w/w), dry
LIGH	0.195	(w/w), dry
LIGO	0.125	(w/w), dry
$\frac{\lambda_{effr}}{\lambda_{effx}}\left(unpyrolized\;pellet\right)$	0.906 (estimated)	-
$\frac{\lambda_{effr}}{\lambda_{effx}}\left(pyrolized\;pellet\right)$	0.917 (estimated)	-
*K*_Gb,x_	1.55 × 10^–15^	m^2^
*K*_Gb,r_	3.74 × 10^–16^	m^2^
*K*_Gc,x_	4.24 × 10^–12^	m^2^
*K*_Gc,r_	2.63 × 10^–12^	m^2^
*s*_x_char_	0.197	
*s*_r_char_	0.320	-

a Weight fractions of cellulose, hemicellulose, and lignin from GC–MS analysis of loblolly pine particles performed at NREL.

#### 3.4.1 Effect of the radial mass transport (radial permeability) on the residual mass prediction from particle modeling

To study the effect of radial permeability on particle conversion, two scenarios were examined: radial mass transport (case 1a) and the absence of radial mass transport (case 2), to emulate a particle with highly anisotropic permeability where the radial mass transport can be considered negligible. Figure 21 shows the residual mass fraction as a function of time for both scenarios. In both cases, the behavior of the initial heating stage was similar up to about 35 s. After this initial heating stage, in case 1a, a lower reaction rate was observed compared to case 2, which accelerated and dropped below the experimental mean, revealing an earlier conversion. The acceleration of the heating rate in case 2 can be explained by the accumulation of internal energy due to the lack of radial transport; the vapors produced therefore remained longer within the biomass matrix, causing the local temperature to rise. This behavior is consistent with the temperature dependence of Arrhenius-type kinetic constants (*k* ∝ $e^{-E_{a}/RT}$ ), leading to an increase in the overall reaction rate. Case 1 provided a better fit, remaining within the experimental error band throughout the simulation and reaching a char yield similar to that observed experimentally. In contrast, case 2 tended to under-predict conversion due to the restriction to the evacuation of gases produced inside the particle, yielding a higher char yield compared to the one measured experimentally.

**Figure 21:**
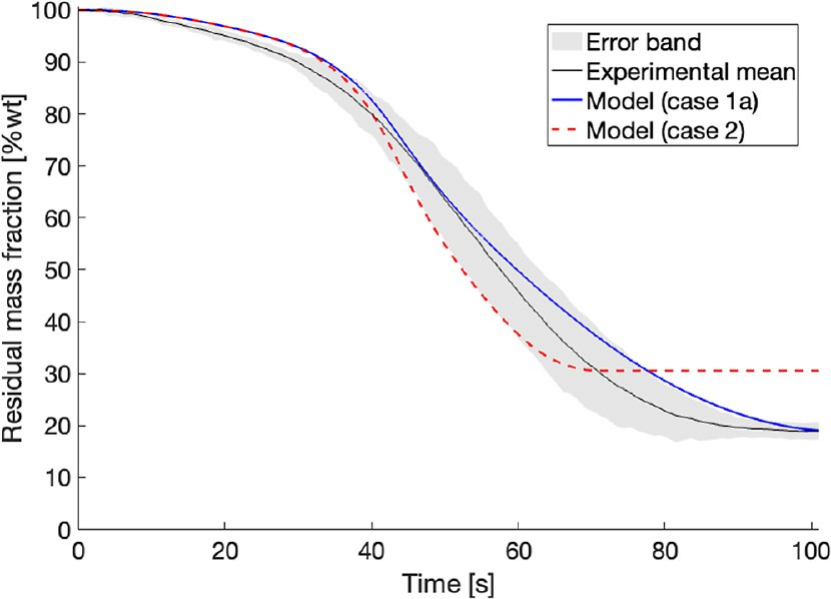
Effect of the radial mass transport on residual mass fraction evolution from particle modeling sensitivity analysis. Case 1a: axial and radial mass transport. Case 2: no radial mass transport.

Figure 22 shows the temperature and pressure contours for cases 1 and 2 at 50 s. The temperature contour for case 1a (Figure 22a) highlights a clear dissipative effect produced by radial mass transport. In case 2 (Figure 22c), by contrast, the temperature contour was relatively uniform because the absence of radial mass transport reduced convective energy transport; heat, therefore, propagated radially by conduction, and gases moved only axially. This led to a more uniform reaction rate throughout the pelletized pine particle and, consequently, an earlier overall conversion. The pressure distribution in Figure 22b (case 1a) shows that internal pressures remained below 1.5 × 10^5^ Pa, consistent with the earlier discussion that gases escaped the pelletized pine sooner when radial transport was present. In case 2 (Figure 22d), local pressures reached up to 10 × 10^5^ Pa because of gas accumulation inside the pelletized pine. This behavior agrees with previous studies indicating that higher pressures shift the system equilibrium toward condensable products and increase char yield.^58^

**Figure 22:**
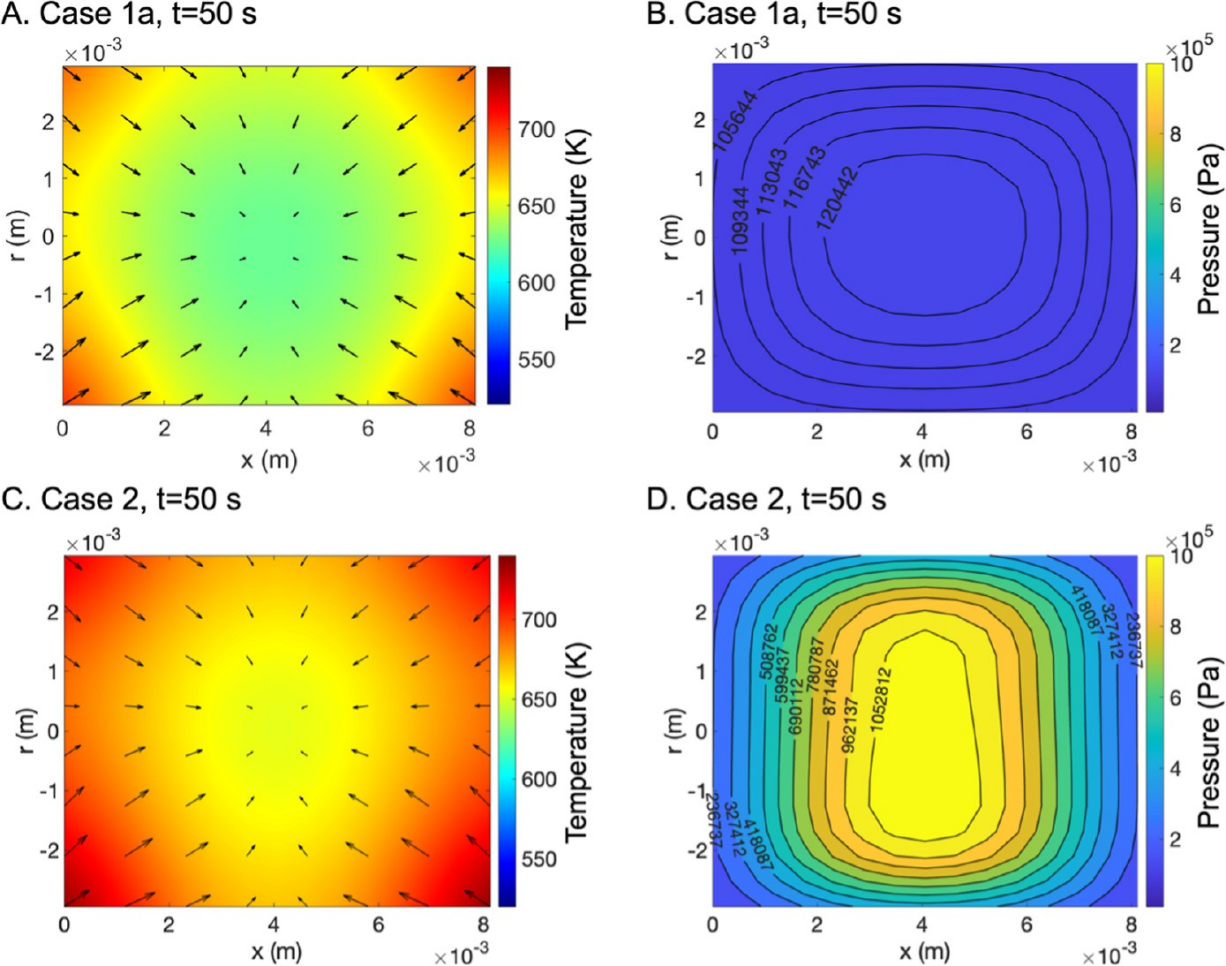
Effect of radial mass transport on temperature and pressure contours (*t* = 50 s): (A) temperature contours for radial mass transport scenario (case 1a), (B) pressure contours for radial mass transport scenario (case 1a), (C) temperature contours for no radial mass transport scenario (case 2), and (D) pressure contours for no radial mass transport scenario (case 2).

#### 3.4.2 Effect of the Thermal Conductivity Anisotropy Ratio

The effect of the thermal conductivity anisotropy ratio was investigated by comparing simulation cases featuring high isotropy, representing pelletized material (case 1a: λ_effr_ = 0.906λ_effx_), and high anisotropy, representing a typical softwood feedstock (case 3: λ_effr_ = 0.580λ_effx_). These results are shown in Figure 23 and demonstrate that directional parametrization of the thermal conductivity in both directions influenced the prediction of the residual biomass fraction. In case 1a, the highly isotropic pelletized pine exhibited a slightly faster conversion rate, which shows that isotropic transfer accelerates conversion in the context of densified biomass. In both scenarios, the residual mass fraction settled at approximately 20%, although different conversion times were found for both scenarios.

**Figure 23:**
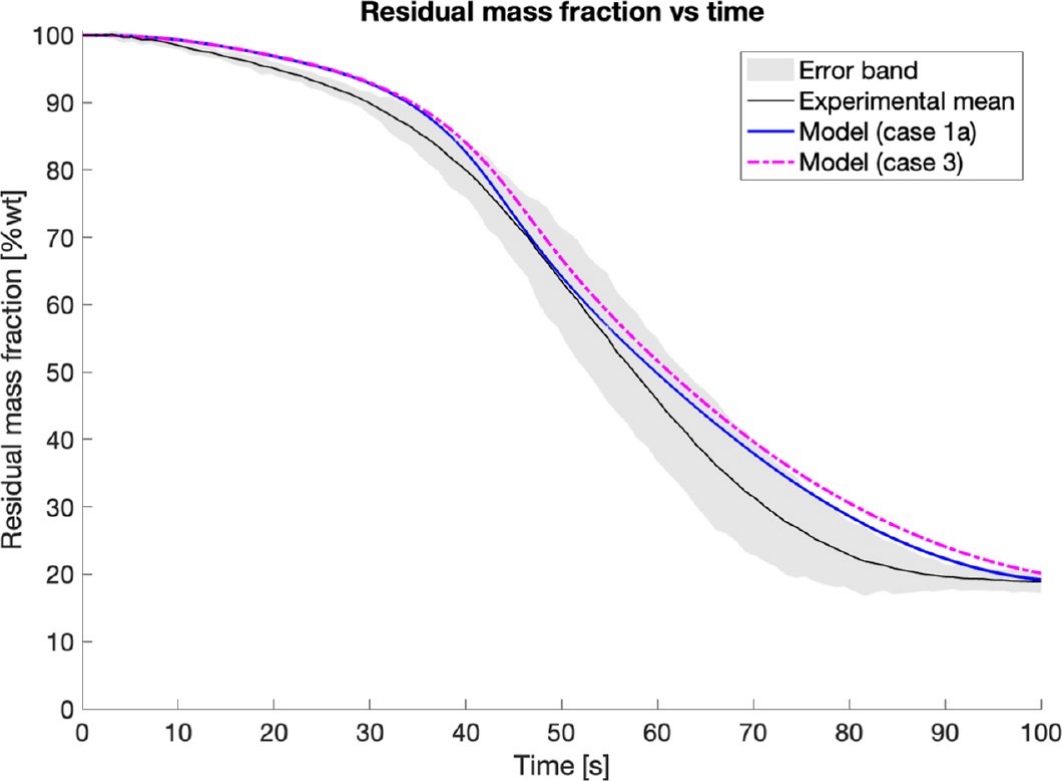
Effect of the pelletized pine thermal conductivity ratio on residual mass fraction evolution from particle modeling sensitivity analysis. Case 1a: nearly isotropic thermal conductivity. Case 3: anisotropic thermal conductivity.

Temperature contours are shown in Figure 24. At 40 s of the process, as shown in Figure 24a, the pelletized pine core in the anisotropic case remained below 580 K, whereas in the isotropic case, temperatures exceeded 600 K, evidencing lower resistance to radial heat transfer. In the anisotropic pelletized pine (case 3), the contours adopted an elliptical shape—highlighting dominant axial conduction—while in the isotropic pelletized pine (case 1a), they were almost circular, indicating a more uniform energy transport. Figure 24b,d at 70 s show a difference of around 20 K at the center of the pelletized pine particle; the higher center temperature for the isotropic case implies a higher conversion rate within the particle.

**Figure 24:**
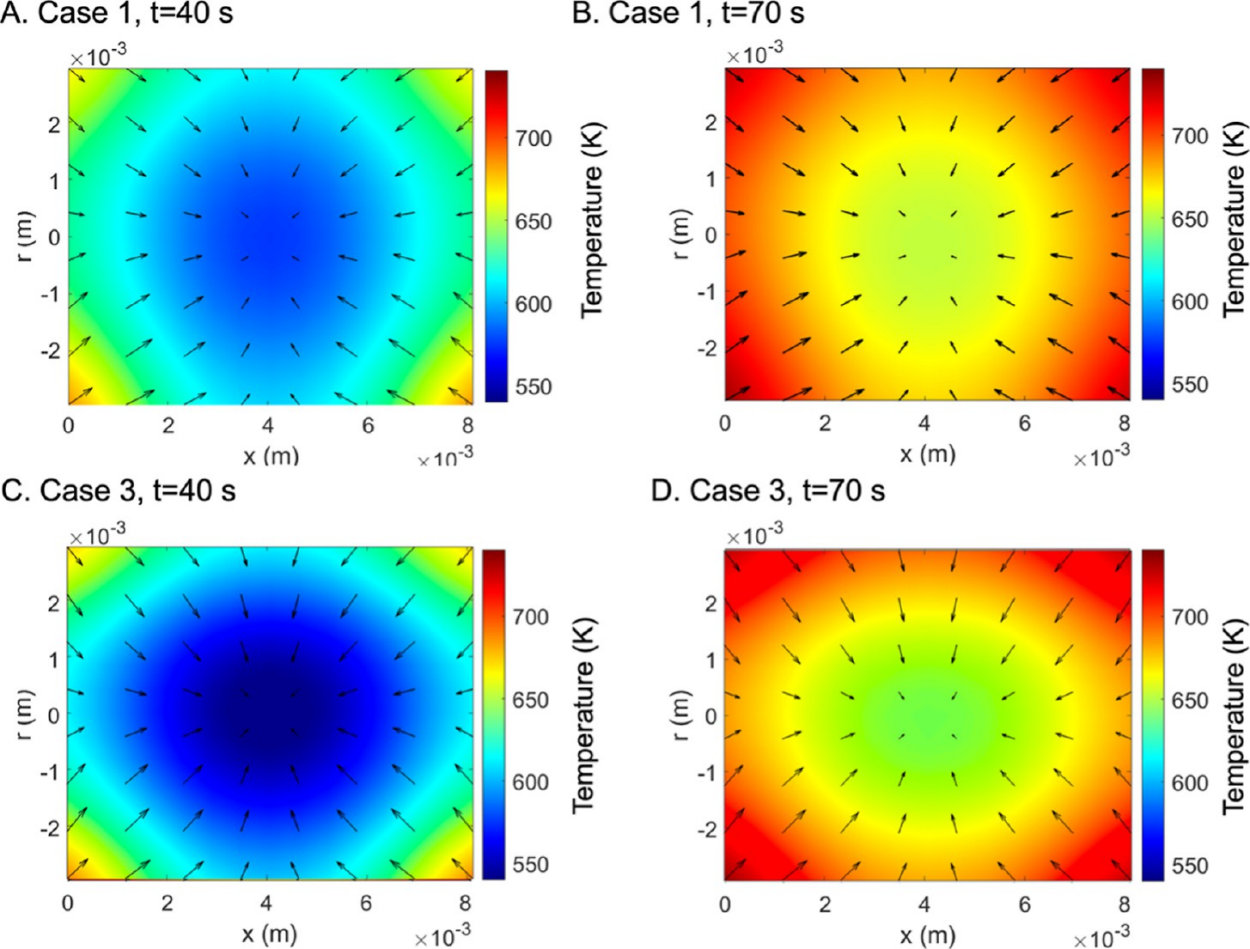
Effect of thermal conductivity anisotropy ratio on temperature contours: (a) temperature contours for highly isotropic particle *t* = 40 s (case 1a), (b) temperature contours for highly isotropic particle *t* = 70 s (case 1a), (c) temperature contours for highly anisotropic particle *t* = 40 s (case 3), and (d) temperature contours for highly anisotropic particle *t* = 70 s (case 3).

The results from the sensitivity analysis of particle models with respect to anisotropic mass transport and thermal conductivity elucidate the importance of convection in conversion during pyrolysis and help to interpret experimentally observed differences in the conversion of cut and pelletized pine. Intraparticle convection occurs in response to pressure building inside the particle as devolatilization proceeds, which forces vapors out of the particle. Since the velocity vector field is directed away from the particle, this convection serves to remove heat from the particle, which opposes conductive heat transfer into the particle. In the case of the pelletized material, restricted convection contributes to a buildup of pressure within the pelletized pine particle, which further favors char production.^58^ The lower temperature experienced by the pelletized pine particle during pyrolysis predicted by the reactor model (Figure S1) also slows conversion and contributes to the preferential formation of char over volatiles. These findings emphasize the importance of incorporating accurate, feedstock-specific transport properties into models for better prediction of the complex transport phenomena, leading to unique conversion behavior during pyrolysis.

## 4 Conclusions

A comprehensive comparison of neat and pelletized pine was presented, consisting of experiments performed using NREL’s SPR, XCT imaging of feedstock particles before and after pyrolysis, and computational modeling. In summary, these analyses revealed that pelletization increased density and tortuosity, decreased porosity and permeability, and ultimately resulted in slower effective conversion and increased char yield relative to neat material. Sensitivity analyses from particle modeling highlight the importance of accurate representation of transport properties to achieve close agreement with experiment. Additionally, our modeling framework aligns well with experimental char yields for pelletized particles using the CRECK reaction mechanism; however, modification of several of the char and metaplastic forming reactions improved the char yield prediction for neat pine. Collectively, the experiments, characterization, and modeling presented in this study provide insight into the effect of wood pelletization and its impact on material properties and transport phenomena and elucidate how these influence effective conversion times and product distributions of fast pyrolysis.

## Supplementary Material



## Data Availability

Data is available on the Bioenergy DataHub, sponsored by the Feedstock Conversion Interface Consortium (FCIC)—https://bioenergy.labworks.org/labkey/FCIC/project-begin.view. Data on the DataHub is in the NREL Single Particle Reactor folder under Task 6—High Temperature Conversion. Data on the feedstock provenance is available on the Biomass Feedstock Library, part of the Biomass Feedstock National User Facility sponsored by the US Department of Energy’s Bioenergy Technologies Office, in the following data set—https://bioenergylibrary.inl.gov/data/dataset.aspx?id=1018. Data is also available upon request to the authors.

## Notes

Proximal analysis and densities of feedstock particles; additional description of XCT analysis; and nomenclature and symbol definitions (PDF)
