# Supplementary material for: Quantifying Impacts of Biomass Pelletization on Fast Pyrolysis Using a Single-Particle Reactor, X‑ray Computed Tomography, and Computational Modeling
Source: Energy Fuels. 2025 Nov 20;40(6):3136–58. doi: 10.1021/acs.energyfuels.5c04362 (PMC12908120; doi:10.1021/acs.energyfuels.5c04362)
Supplement: Supplementary file 1 [file ef5c04362_si_001.pdf]

Supporting Information for:

Quantifying Impacts of Biomass Pelletization on Fast Pyrolysis using a Single Particle Reactor, X-ray Computed Tomography, and Computational Modeling

Meagan F. Crowley,[1‡] Reinhard Seiser,[1‡] Mario Alejandro Sánchez Posada,[2‡] Juan C. Maya,[3] Farid Chejne,[3] Hariswaran Sitaraman,[1] Francois Usseglio-Viretta,[1] Anne K. Starace,[1] Peter N. Ciesielski[1*]

[1]National Renewable Energy Laboratory (NREL), Golden, CO 15013, USA

[2]Grupo de Investigación ENERGEIA, Escuela de Ingeniería y Ciencias Básicas, Universidad EIA, Envigado 055428, Colombia

[3]Grupo de Investigación TAYEA, Departamento de Procesos y Energía, Facultad de Minas, Universidad Nacional de Colombia-Sede Medellín, Medellín 050034, Colombia

‡These authors contributed equally.
*Address correspondence to: peter.ciesielski@nrel.gov

Table S1. Properties of feedstocks. Provenance data can be found on the Biomass Feedstock Library, part of the Biomass Feedstock National User Facility sponsored by the US Department of Energy's Bioenergy Technologies Office, in the following dataset: https://bioenergylibrary.inl.gov/data/dataset.aspx?id=1018.

Feed-stock	GUID (Biomass Feedstock Library)	Density (g/m^3)	C (weight %)	H (weight %)	N (weight %)	Moisture (weight %)	Volatile Matter (weight %)	Fixed carbon (weight %)	Ash (weight %)
Cut Pine	f4c58c4c-7495-6b42-b0a5-1fc9bf4d89c	0.5 to 0.6	46.99	6.65	0.19	7.21	77.78	14.92	0.09
Pelletized Pine	069a649a-e17f-4f7c-a96e-b76f6b5f710f	1.1 to 1.2	49.15	6.61	Below detectable limit	4.66	83.57	11.09	0.68

Table S2. Bulk density of biomass samples used for determining threshold for XCT sub volumes.

Sample	Average diameter (mm)	Average length (mm)	Mass (g)	Bulk density (kg/m^3)
Cut pine	6.30 ± 0.05	7.86 ± 0.04	0.152 ± 0.001	620 ± 10
Cut pine char	4.2 ± 0.2	6.66 ± 0.04	0.026 ± 0.001	280 ± 15
Pelletized pine	5.88 ± 0.02	8.12 ± 0.03	0.274 ± 0.001	1239 ± 8
Pelletized pine char	4.00 ± 0.05	6.51 ± 0.09	0.048 ± 0.001	590 ± 14

The diameter and length of each sample were measured using calipers and the mass was measured using an analytical balance. The volume was calculated assuming a cylindrical particle based on the average measured diameter and length. The bulk density of each particle was calculated as the ratio of the average measured mass to the calculated volume. Measurements of length, diameter, and mass were each performed five times on each sample and the average and standard deviation are reported in Table S2. The error of the bulk density was calculated from the propagated error in length, diameter, and mass measurements.

The threshold for segmenting the XCT sub-volumes into solid and void phases was chosen to yield a calculated porosity which reproduced the bulk density of each sample, according to Equation S1:

$$\varepsilon = 1 - \frac{\rho_{bulk}}{\rho_{sk}} \ (S1)$$

Where ε is the porosity of the sample, ρ_{bulk} is the bulk density of each sample reported in Table S2, and ρ_{sk} is the skeletal density of the biomass material: 1500 kg/m^3 for cut

pine and pelletized pine and 1700 kg/m^3 for cut pine char and pelletized pine char. The densities of the sub-volumes using the determines thresholds matched the bulk density of each sample within 8%.

Table S3. Nomenclature

**Symbol**	**Description**	**Units**
acqua	Liquid water (moisture)	
b	biomass	
c	char	
CELL	Cellulose	
CELLA	Active cellulose	
C_p	Particle local heat capacity	J·kg^{-1}K^{-1}
$C_{p,i}$	heat capacity of the gaseous components	J·kg^{-1}K^{-1}
D	Particle diameter	m
D_{eff}	Effective diffusivity of species in the gas phase	m^2·s^{-1}
$E_{a,j}$	Activation energy for the reaction *j*	kJ·mol^{-1}
G	Gas phase (permanent gases, volatiles, water vapor)	
GAS	Permanent gases	
h	External convective heat transfer coefficient	W·m^{-2}·K^{-1}
HCE	Hemicellulose	
HCE1	Active hemicellulose type 1	
HCE2	Active hemicellulose type 2	
H_2O	Water vapor	
H_{R_j}	Heat of reaction for reaction *j*	kJ·kg ^{1}
i	Species or the reaction mechanism	
j	Reactions of the reaction mechanism	
$k_{0,j}$	Kinetic coefficient for the reaction j	s^{-1}
k_j	Kinect rate for the reaction *j*	s^{-1}
$k_{m,i}$	External convective mass transfer coefficient	kg·s^{-1}·m^{-2}
K_G	Solid permeability to the gas mixture flow (axial direction)	m^2
$K_{G, b}$	Biomass permeability to the gas mixture flow (axial direction)	m^2
$K_{G, char}$	Char permeability to the gas mixture flow (axial direction)	m^2
L_0	Initial particle length	m
L_f	Final particle length	m
L	Liquid intermediate phase (metaplast phase)	
LIG-C	Carbon riched lignin	
LIG-CC	Intermediate component from lignin	
LIG-H	Hydrogen riched lignin	
LIG-O	Oxygen riched lignin	
LIG-OH	Intermediate component from lignin	
LIG	Intermediate component from lignin	
M_i	Molecular weight of the specie *i*	kg·mol^{-1}
N_r	Total number of reactions	
$\dot{N}_x''$	Mass flux in the axial direction	kg·m^{-2}·s^{-1}
P	Pressure of the gas mixture	Pa
$\dot{q}_x$	Heat flux in the axial direction	W·m^{-2}
$\dot{q}_r$	Heat flux in the radial direction	W·m^{-2}
$\dot{r}'''_{j,i}$	Reaction rate for the specie *i* in the reaction *j*	
R_0	Initial particle radius	m
R_f	Final particle radius	m
R_u	Universal constant of ideal gases	J·mol^{-1}K^{-1}
S	Solid phase	
$s_{z,f}$	Final shrinking coefficient in the axial direction	
$s_{r,f}$	Final shrinking coefficient in the radial direction	
t	time	s^{-1}
T	Temperature	K

T_{prom}	Particle average temperature	K
T {CO2}	CO_2 trapped in the metaplast phase	
V	Volume of the particle	m^3
VOL.	Volatiles (light condensables)	
w_i	Weight fraction of the specie *i*	
Greek symbols		
α_i	Molar stoichiometric coefficients of the specie *i*	
γ_i	Molar stoichiometric coefficients	
ε_i	Component/phase fraction	
$\bar{\eta}$	Average conversion of the particle	-
$\lambda_{i,x}$	Thermal conductivity of the component/solid in the axial direction	$W\cdot m^{-1}\cdot K^{-1}$
$\lambda_{i,r}$	Thermal conductivity of the component/solid in the radial direction	$W\cdot m^{-1}\cdot K^{-1}$
$\lambda_{eff,x}$	Effective conductivity in the axial direction	$W\cdot m^{-1}\cdot K^{-1}$
$\lambda_{eff,r}$	Effective conductivity in the radial direction	$W\cdot m^{-1}\cdot K^{-1}$
λ_{Π}	Theoretical conductivity in the axial direction	$W\cdot m^{-1}\cdot K^{-1}$
$\lambda_{\perp}$	Theoretical conductivity in the radial direction	$W\cdot m^{-1}\cdot K^{-1}$
μ_g	Viscosity of the gas mixture	$Pa\cdot s$
ξ	Relation between axial and radial effective thermal conductivity	
ρ	Local particle density (estimated for each node)	$kg\cdot m^{-3}$
ρ_i	Mass concentration of the component *i* per phase volume	$kg\cdot m^{-3}$
ρ_i^*	Mass concentration of the component *i* per particle volume	$kg\cdot m^{-3}$
$\rho_{i_g}^*$	Mass concentration of the component *i* in the gas phase per particle volume	$kg\cdot m^{-3}$
$\bar{\rho}_i$	Component intrinsic density	$kg\cdot m^{-3}$
ρ_∞^*	Concentration of the specie *i* in the bulk phase	
$\rho_{R,j}^*$	Concentration of the reactant that produces the specie *i* in the reaction *j*	$kg\cdot m^{-3}$
v_g	Velocity of the gas mixture	$m\cdot s^{-1}$

Table S4. Modified activation energies for cut pine particle model. Original kinetic parameters are reported in Debiagi et al, 2018 (27)

Reaction number	Equation	Activation energy (J/mol)
4	CELL -> 0.125H2 + 4.45H2O + 5.45CHAR + 0.12G{COH2}Stiff + 0.25G{CO} + 0.18G{COH2}Loose + 0.125G{H2}	840.7E3
9	HCE1 - > 0.4CH2O + 0.49CO + 0.39CO2 + 0.1H2 + 0.4H2O + 0.05HCOOH + 0.1C2H4 + 0.3CH4 + 0.975CHAR + 0.37G{COH2}Stiff + 0.51G{CO2} + 0.01G{CO} + 0.325G{CH4} + 0.075G{C2H4} + 0.43G{COH2}Loose + 0.05G{H2} + 0.2G{C2H6}	30E3
17	LIG -> 0.4CH2O + 0.3CO + 0.1CO2 + 0.6H2O + 0.2CH4 + 6.1CHAR + 0.65G{COH2}Stiff +	643E3

	0.2G{CO} + 0.4G{CH3OH} + 0.4G{CH4} + 0.5G{C2H4} + 1.25G{COH2}Loose + 0.1G{H2}	

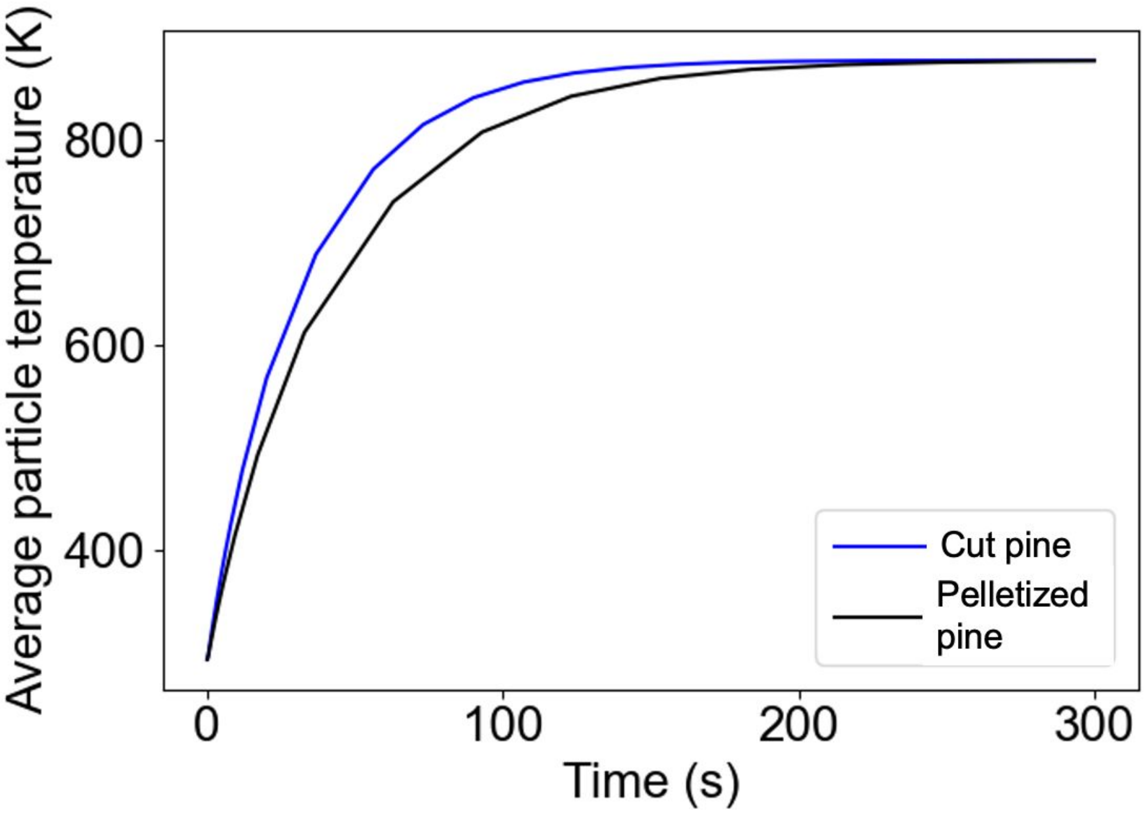

Figure S1. Volume averaged particle temperature vs. time predicted by the reactor scale model for pelletized pine and cut pine
